# Supplementary material for: Causal associations between risk factors and common diseases inferred from GWAS summary data
Source: Nat Commun. 2018 Jan 15;9:224. doi: 10.1038/s41467-017-02317-2 (PMC5768719; doi:10.1038/s41467-017-02317-2)
Supplement: Supplementary file 1 — Supplementary Information [file 41467_2017_2317_MOESM1_ESM.pdf]

Supplementary Note 1: Simulation studies

We conducted a series of simulations to investigate 1) whether there is inflation in GSMR test-statistic under the null hypothesis, 2) the unbiasedness of GSMR estimate of b_{xy} under the alternative hypothesis, 3) the interpretation of b_{xy}, 4) power of GSMR, 5) power of HEIDI-outlier test and unbiasedness of $\hat{b}_{xy}$ in the presence of pleiotropy, and 6) the test statistic of reverse-GSMR.

To investigate whether there is inflation in GSMR test-statistic under the null hypothesis

We performed simulations to investigate whether there is inflation in GSMR test-statistic under the null hypothesis that there is no causal association between exposure (x) and outcome (y) but x and y are associated due to a non-genetic confounding factor. We simulated the genotypes of 100 SNPs from a binomial distribution $z \sim B(2, f)$ with f being the allele frequency of a SNP, $f \sim U(0.01, 0.5)$. The exposure was simulated based on the model $x = zb_{zx} + c + e_{zx}$, where c is the latent non-genetic confounding variable with $c \sim N(0, \sigma_c^2)$, $\sigma_c^2 = \text{var}(zb_{zx})R_{cx}^2/R_{zx}^2$, R_{cx}^2 is the proportion of variance in x explained by c ($R_{cx}^2 = 0.5$), R_{zx}^2 is the proportion of variance in x explained by z ($R_{zx}^2 = 0.15$), and $e_{zx} \sim N[0, \text{var}(zb_{zx})\left(\frac{1}{R_{zx}^2} - 1\right) - \sigma_c^2]$. We generated b_{zx} from $N[0, \frac{1}{2f(1-f)}]$. The outcome was simulated based on the model $y = c + e_y$, where $e_y \sim N[0, \sigma_c^2\left(\frac{1}{R_{cy}^2} - 1\right)]$ with R_{cy}^2 being the proportion of variance in y explained by c (R_{cy}^2 = 0.5). Note that if x and y are measured in the same sample, x will appear to be associated with y due to the confounding factor c. To mimic a two-sample MR analysis, we simulated 50,000 individuals as sample #1 (n_1) and 20,000 individuals as sample #2 (n_2). We then performed simple regression analysis to estimate b_{zx} in sample #1 and b_{zy} in sample #2. In addition, we simulated 5,000 individuals as sample #3 (n_3) to calculate LD correlation matrix. Each simulation was replicated 1,000 times. For simplicity but without loss of generality, we used 100 SNPs in the simulations below.

To investigate the unbiasedness of GSMR estimate of b_{xy} under the alternative hypothesis

SNP instruments are independent: We simulated x using the same method as above with three levels of R_{zx}^2 (0.15, 0.2 and 0.3) and generating outcome based on the model $y = xb_{xy} + c + e_{xy}$, where b_{xy} is the causal effect of x on y ($b_{xy} = 1$) and $e_{xy} \sim N(0, \sigma_{e(xy)}^2)$ with $\sigma_{e(xy)}^2 = \text{var}(xb_{xy})\left(\frac{1}{R_{xy}^2} - 1\right) - \sigma_c^2 - 2\text{cov}(xb_{xy}, c) = \text{var}(xb_{xy})\left(\frac{1}{R_{xy}^2} - 1\right) - \sigma_c^2(1 + 2b_{xy})$ and R_{xy}^2 is proportion of variance in y explained by x with R_{xy}^2 = 0.05 and 0.1. The estimates of b_{xy} from 1,000 simulation replicates are shown in **Supplementary Table 1**.

SNP instruments are in LD: We extracted SNP genotypes of a 15Mb segment on chromosome 22 from the UKB data. After pruning SNPs for LD with a LD r^2 threshold of 0.5 and a window size of 100kb, 2,006 SNPs were retained. We then randomly sampled 100 SNPs as causal variants, and simulated x and y using the same method as above. GSMR and weighted generalized linear regression (i.e., generalized MR-IVW) were used to estimate b_{xy}. We randomly sampled 50,000 individuals as sample #1 and 20,000 individuals as sample #2. For data analysis, the LD correlations among SNPs were estimated from a reference sample (sample #3) of 5,000 individuals randomly sampled from the ARIC data. The estimates of b_{xy} from 1,000 simulation replicates are shown in **Supplementary Table 1**.
The effect of sample size for the exposure on the unbiasedness of GSMR: We simulated x and y using the same method as above with $R_{zx}^2 = 0.15$, $R_{xy}^2 = 0.05$, and the sample size of sample #1 varying from 50,000 to 200,000 with an increment of 25,000. The mean estimates of b_{xy} from 1,000 simulation replicates are shown in **Supplementary Figure 20.**

To investigate whether we can interpret b_{xy} as logOR

We generated x and y under alternative hypothesis that x has a causal effect on y using the same method as described above with $R_{zx}^2 = 0.15$, $R_{zy}^2 = 0.05$, n_1 = 50,000 and n_2 = 2,000,000. Note that in order to demonstrate the equivalence between the estimate of b_{xy} from GSMR and logOR from logistic regression we did not simulate a confounding factor in this case. In sample #2, we transformed y to a dichotomous phenotype (y_{01} = 1 for affected and 0 for unaffected) based a liability-threshold model. The disease prevalence (k) was simulated from $U(0.005, 0.01)$. We then randomly sampled 10,000 cases (affected) and 10,000 controls (unaffected) from the entire sample. We estimated logOR by logistic regression of y_{01} on x in sample #2. The GSMR estimate of b_{xy} was obtained using the summary data from the two samples. The simulation was performed with 1,000 replicates. A comparison between the estimates of b_{xy} using two-sample GSMR and logOR using one-sample logistic regression from 1,000 simulation replicates is shown in **Supplementary Fig. 2**.

To investigate the statistical power of GSMR

We simulated 1,000 independent SNPs from a binomial distribution $z \sim B(2, f)$. We then generated x and y under the assumption that x has a causal effect on y, using the same method as above with $R_{zx}^2 = 0.6$, $R_{xy}^2 = 0.005$, n_1 = 50,000 and n_2 = 20,000. We conducted analyses using GSMR, MR-Egger[1], MR-IVW[2], generalized MR-IVW[3], and the Pickrell methods[4] based on a random subset of the genome-wide significant SNPs associated with x, the number of instruments $m \sim U(10, m_t)$ where m_t is the total number of genome-wide significant SNPs. We ran the simulations with 10,000 replicates.

To investigate the statistical power of HEIDI-outlier and the unbiasedness of GSMR estimate of b_{xy} in the presence of pleiotropy

SNP instruments are independent: We simulated genotypes of 550 SNPs (z) from a binomial distribution $z \sim B(2, f)$, including 500 SNPs (z_c) that have effects on *y* mediated by *x*, and 50 SNPs (z_p) that have horizontal pleiotropic effects on both *x* and *y*. We simulated *x* using a similar model as above $x = z_c b_{z_c x} + z_p b_{z_p x} + c + e_{zx}$

where $e_{zx} \sim N[0, \text{var}\left(z_c b_{z_c x} + z_p b_{z_p x} + c\right)\left(\frac{1}{R^2_{z_c x} + R^2_{z_p x} + R^2_{cx}} - 1\right)]$. All the effect sizes were generated from $N[0, \frac{1}{2f(1-f)}]$ where *f* is the allele frequency. We set $R^2_{z_c x} = 0.4$, $R^2_{cx} = 0.2$ and $R^2_{z_p x} = R^2_{z_p}$ with $R^2_{z_p}$ varying from 0.03 to 0.1 with an increment of 0.01. The outcome *y* was simulated based on the model

$$y = x b_{xy} + z_p b_{z_p y} + c + e_y$$

where $e_{xy} \sim N(0, \sigma^2_{e(xy)})$ with $\sigma^2_{e(xy)} = \text{var}\left(x b_{xy} + z_p b_{z_p y} + c\right)\left(\frac{1}{R^2_{xy} + R^2_{z_p y} + R^2_{cy}} - 1\right) - \sigma^2_c\left(1 + 2b_{xy}\right)$. We set b_{xy}=1, $R^2_{xy} = 0.05$ and $R^2_{z_p y} = R^2_{z_p}$. We simulated 50,000 individuals as sample #1, 20,000 individuals as sample #2 and 5,000 individuals as sample #3. We performed the HEIDI-outlier analysis to quantify the power of detecting pleiotropic SNPs. We then estimated b_{xy} by GSMR (after HEIDI-outlier filtering) and MR-Egger (without HEIDI-outlier filtering) to quantify the (un)biasedness of the two methods. The simulation was repeated 1,000 times. The results are presented in the **Supplementary Figure 4**. We also investigated the (un)biasedness of $\hat{b}_{xy}$ under a pleotropic model by setting b_{xy} to zero. The results from 1,000 simulation replicates are shown in **Supplementary Table 2**.

SNP instruments are in LD: We extracted SNP genotypes from the UKB data following the strategy described above, and simulated *x* and *y* using the same models as above. Samples #1 (n_1=50,000) and #2 (n_2=20,000) were from the UKB data, and sample #3 were from the ARIC data (n_3=5,000). We investigated the (un)biasedness of GSMR in estimating b_{xy} under a pleotropic model in 1,000 simulation replicates. The results are presented in the **Supplementary Table 2**.

To investigate the test statistic of reverse-GSMR

We simulated *x* based on 110 simulated SNPs (z) from a binomial distribution $z \sim B(2, f)$, including 100 SNPs (z_c) that have effects on *y* mediated by *x*, and 10 SNPs (z_r) that have direct effects on *y*. We simulated *x* based on the same model as above with $R^2_{z_c x} = 0.15$ and $R^2_{cx} = 0.5$. We then simulated *y* based on a model similar to that described above

$y = xb_{xy} + z_r b_{z_r y} + c + e_y$

where $e_y \sim N(0, \sigma_{e_y}^2)$ with $\sigma_{e_y}^2 = \mathrm{var}(xb_{xy} + z_r b_{z_r y})\left(\frac{1}{R_{xy}^2 + R_{z_r y}^2} - 1\right) - \sigma_c^2(1 + 2b_{xy})$ and $R_{z_r y}^2$ is the variance in *y* explained by z_r. We set $R_{z_r y}^2 = 0.1$ and $R_{xy}^2 = 0.1$. *P*-values from the reverse-GSMR analysis (using *y* as the exposure) with 1,000 replicates are shown in **Supplementary Figure 19**.

Supplementary Note 2: Multi-trait-based conditional and joint GWAS analysis using summary data (mtCOJO)

To investigate the accuracy of mtCOJO

We performed simulations to investigate the accuracy of the approximate multi-trait based conditional and joint (mtCOJO) association analysis. The simulation was conducted in the imputed UKB data after QC (**Methods**). For the ease of computing, we only included the SNPs in common with HapMap phase 3. SNPs were partitioned into 2 subsets, those on the odd chromosomes and even chromosomes. Causal variants as described below were sampled from the even chromosomes. We assume that there were 3 correlated traits, x_1, x_2 and x_3. We randomly sampled 600 SNPs (z) as SNP set #1 and generated x_1 based on the model $x_1 = \sum_i z_i b_{zx1(i)} + e_{x1}$ with $R_{zx1}^2 = 0.5$, where R_{zx1}^2 is the proportion of variance in x_1 captured by all the SNPs. We then randomly sampled 600 SNPs as SNP set #2 of which 300 SNPs were in common with those in SNP set #1 (with the same effect sizes). Note that in the pleotropic model below only 200 SNPs are in common between x_1 and x_2, and these 200 SNPs have the same effects on x_1 and x_2. The effect sizes were generated from the standard normal distribution. We generated x_2 using the same method as above with $R_{zx2}^2 = 0.5$. We simulated x_3 based on two different models: 1) causality $x_3 = x_1 b_{x1x3} + e_{x1x3}$ with $R_{x1x3}^2 = 0.2$, where b_{x1x3} is the effect size of x_1 on x_3 ($b_{x1x3} = 1$) and R_{x1x3}^2 is the variance in x_3 explained by x_1; 2) type-II pleiotropy $x_3 = \sum_i z_i b_{zx3(i)} + e_{zx3}$, where x_3 has 200 SNPs in common with x_1, another distinct set of 200 SNPs in common with x_2 (no overlap with the SNPs above) and an additional set of 200 variants randomly sampled from the rest SNPs in UKB. We then conducted GWAS analyses for the three traits and estimated effect sizes of z on x_3 conditional on genetic value of x_1 and x_2 (g_{x1} and g_{x2}) using the mtCOJO approach as described in **Methods** (based only on summary data) and the exact approach (regressing x_3 on z, g_{x1} and g_{x2}, where g_{x1} and g_{x2} could be observed in the simulations). The GSMR instruments were selected by clumping analysis (r^2 threshold = 0.05, p-value threshold = 5e–8, and window size = 5Mb). The ARIC data was used as the reference sample to estimate LD between each pair of instruments. A comparison between the estimates of $\hat{b}_{zx_3}|(\hat{b}_{x_1x_3}, \hat{b}_{x_2x_3})$ using the mtCOJO approach and the exact is shown in **Supplementary Figure 5**.

We further investigated whether the test-statistics are inflated or not when there are differences in the sample sizes between x_1, x_2 and x_3. Under a causal model, we would not expect to see inflation because there would not be remaining effect of SNPs on x_3 conditioning on g_{x1} and g_{x2} for SNPs on even chromosomes, and we would also not expect to see inflation for the null SNPs on odd chromosomes. To do this, we performed GWAS analysis for x_1 and x_2 using subsets of individuals, n = 30,000 for x_1 and 20,000 for x_2 without sample overlap. We the performed GWAS for x_3 1) using a subset of individuals (n = 50,000) without sample overlap with x_1 and x_2, and 2) using all the individuals in the whole sample (n = 108,039). The QQ plot of p-values for $\hat{b}_{zx_3}|(\hat{b}_{x_1x_3}, \hat{b}_{x_2x_3})$ is shown in **Supplementary Figure 5**.

To quantify the bias described in Aschard et al. (AJHG 2015)

To investigate whether the estimate of conditional SNP effect from mtCOJO is suffered from the bias described in Aschard *et al.*[5] or not, we performed simulation under the scenario described in their Figure 1c that there is no direct effect of the SNP in question on the phenotype but the phenotype and covariate are correlated due to shared environmental or genetic factors. We randomly sampled 600 SNPs (z_c) as the confounding genetic factors from the odd chromosomes and simulated a phenotype y using a model similar to that described above, i.e. $y = \sum_i z_{c(i)} b_{z_c y(i)} + e_y$. The variance in y explained by z_c ($R^2_{z_c y}$) was 0.3. We then randomly sampled 600 SNPs (z) from the even chromosomes (therefore independent of z_c), simulating a covariate x based on the model $x = \sum_i z_{c(i)} b_{z_c x(i)} + \sum_j z_i b_{zx(j)} + e_x$. The variance in x explained by z_c and z were both 0.3, i.e., $R^2_{z_c x} = R^2_{zx} = 0.3$. All the effect sizes were generated from $N[0, \frac{1}{2f(1-f)}]$ where f is the allele frequency. Under this simulation model, the direct effect of z on y is expected to be zero. However, since y and x are correlated due to the shared genetic factors (z_c) that are independent of the SNPs to be tested (z), the estimate of the effect of z on y conditional on x from a linear regression analysis using individual-level data is expected to be biased[5]. The estimated $\hat{b}_{zy}$ conditional on x from mtCOJO using summary-level data should be unbiased because mtCOJO uses GSMR to distinguish causal effect from confounding effect (**Methods**). The simulation results are presented in **Supplementary Figure 6**.

Supplementary Note 3: Computing logOR and SE from z-statistic from a case-control GWAS

We know that the sampling variance (SE^2) of the estimate of logOR from a logistic regression analysis[6] is approximately 1 / [v(1-v)×$n$var(x)], where v is the proportion of cases in the sample, x is the genotype indicator variable for a SNP (coded as 0, 1 or 2), and n the sample size. Assuming Hardy-Weinberg equilibrium, var(x) = 2f(1-f) where f is the allele frequency, which

can be obtained from a reference sample with individual-level data. We therefore have $SE \approx 1 \Big/ \sqrt{v(1-v) \times 2nf(1-f)}$. If z-statistic is given, we can further have $\log \mathrm{OR} \approx z \Big/ \sqrt{v(1-v) \times 2nf(1-f)}$.

Supplementary Note 4: The effects of WHRadjBMI on common diseases

We included in the analysis two obesity-related traits, body mass index (BMI) and waist-to-hip raio adjusted for BMI (WHRadjBMI). BMI is a measure of the amount of tissue mass and WHRadjBMI is a measure of body fat distribution. Previous studies suggest that BMI- and WHRadjBMI-associated genetic loci are enriched for genes expressed in central nervous system[7] and adipose tissue[8], respectively, which implies potentially different genetic aetiologies of the two traits. We found that the effects of WHRadjBMI and BMI on disease were largely concordant (**Supplementary Fig. 10a**), suggesting that the genetic etiologies of the two traits may differ but their effects on health outcomes are similar. Note that WHRadjBMI has been adjusted for BMI so that that effect sizes of WHRadjBMI on diseases should be independent of BMI. Nevertheless, the effect sizes of WHRadjBMI on diseases were smaller than those of BMI, and WHRadjBMI was detected with significant effects on only 4 diseases, type-2 diabetes (T2D), dyslipidemia, hypertension and coronary artery disease (CAD) (**Fig. 2**), although the smaller number of detections could be because of the smaller number of instruments used for WHRadjBMI (m=43) than for BMI (m=84) as the power of GSMR is proportional to the number of instruments (**Supplementary Fig. 3**).

Supplementary Note 5: The effect of height on common diseases

Results showed that height had significant protective effects against irritable bowel syndrome (OR=0.82), dyslipidemia (OR=0.84), osteoporosis (OR=0.85), acute reaction to stress (OR=0.85), hypertensive disease (OR=0.86), T2D (OR=0.86) and asthma (OR=0.90) in the community data, and CAD (OR=0.77), T2D (OR=0.84), Alzheimer's disease (AD, OR=0.85) and aged macular degeneration (AMD, OR=0.91) in the case-control data (**Fig. 6** and **Supplementary Table 12**). It is interesting to note that height was protective against AD which was not affected by any of the 7 health risk factors. Results also showed that height had a risk effect on varicose veins (OR=1.38), dermatomycosis (OR=1.24), peripheral vascular disease (PVD, OR=1.15), cancer (OR=1.09), osteoarthritis (OR=1.09) and cardiovascular disease (CVD, OR=1.07) in the community data, and rheumatoid arthritis (RA, OR=1.29) in the case-control data. The inconsistent effects of height on CAD (OR=0.77) and CVD (OR=1.07) implies heterogeneous effects of height on vascular outcomes consistent with results from some of the observational studies[9, 10, 11] and a previous Mendelian randomization (MR) study[12]. Height was the only risk

factor that showed a significant risk effect on cancer (P_{GSMR}=3.1×10^{-7}) in this study, in line with the results from previous observational and MR studies[13, 14].

Supplementary Note 6: The association between EduYears and autism spectrum disorder

Since autism spectrum disorder (ASD) is mostly diagnosed in childhood prior to completion of education, the risk effect of educational attainment (EduYears) on ASD is probably because EduYears is a genetic proxy for IQ (r_g=0.74, SE=0.032). While ASD is associated with cognitive deficits in executive function, the relationship with IQ is complex[15]. Our results are consistent with those from a Danish study of more than 160,000 male conscripts[16] which found that brothers of those with ASD had a significantly *higher* than average IQ score (whereas brothers of those with every other recorded psychiatric disorder had significantly lower than average IQ scores). We note that GSMR analysis tests the effect of EduYears on ASD rather the reverse direction (there were not sufficient ASD instruments to perform the reverse GSMR analysis; see below). It is possible that the effects are bi-directional and opposite (see the Results section in the main text for such examples from the reverse GSMR analysis), suggesting a U-shaped relationship between ASD and cognition.

Supplementary Note 7: Reverse-GSMR analysis

We only performed the reverse GSMR analysis for risk factors and diseases showed significant causative associations in the forward GSMR analyses. There were 73 significant associations between the risk factors and diseases (**Supplementary Data #1 and Supplementary Table 12).** We selected independent genome-wide significant SNPs from the disease GWAS data using the PLINK clumping approach (r^2 threshold = 0.05, window size = 1Mb and p-value threshold = 5×10^{-8}; LD from the 1000G-imputed ARIC data). We did not include in the analysis diseases for which the number of instruments was smaller than 10. There were 49 disease and risk factor pairs included in the analysis in total. We then performed the HEIDI-outlier analysis to remove pleiotropic outliers, and run the reverse GSMR analysis to detect effects of diseases on risk factors. The threshold p-value corrected for multiple testing was 1.0×10^{-3} at a family-wise error rate (FWER) of 0.05. The results are shown in **Supplementary Table 13**.

Supplementary Note 8: Caveats in interpreting the GSMR results

First, if the exposure is a composite trait that comprises multiple sub-phenotypes, we could not rule out the possibility that the effect of exposure on disease is driven by one of the sub-phenotypes. For instance, we have identified from the GSMR analysis that EduYears had effects on many diseases (**Fig. 6**). A conservative interpretation is that these are the effects of the genetic component of EduYears (e.g. cognitive ability and personality) on health outcomes. If we express EduYears=g + e where g is the genetic component of EduYears and e is the residual component that includes environmental influence, then the SNPs identified from GWAS for EduYears are those associated with g rather than e, meaning that the GSMR analysis for EduYears was performed on g rather than e and thus did not provide any evidence whether e also has effects on diseases. Therefore, strictly speaking, the causative associations identified in this study are not definitive and need to be confirmed by follow-up randomized controlled trials (RCTs) in the future, if practical. Second, the effect of a risk factor on disease can be non-linear (e.g. the relationship between BMI and mortality is a U-shaped curve[17], suggesting that both underweight and overweight are risk factors of death) whereas we used a linear approximation to estimate the effect because of the limited information that we had access to from GWAS summary data. Therefore, the b_{xy} estimates need to be interpreted with caution at extremes. Third, although we have identified a large number of associations, we would expect that associations of small effect size would be missed in our study (e.g. the instrument for SBP, SBP, was based on only 28 SNPs). The power can be improved in the future with GWAS results based on larger sample sizes. Fourth, our analyses ignored age-specific and sex-specific effects because of the lack of data from age- and sex-stratified analyses. Lastly, we have shown in a previous study that the SMR test-statistic is slightly deflated due to the use of a Taylor series expansion to

compute an approximated sampling variance based on summary data, especially if the association between the instrument and risk factor is not strong enough. We therefore strongly recommend that only SNPs that are associated with the exposure at a genome-wide significance level (i.e. 5×10^{-8}) should be used in GSMR analysis, and as a rule of thumb advise application only when there are 10 or more independent (e.g. $r^2 < 0.05$) genome-wide significant SNPs.

Supplementary Note 9: Acknowledgements

ARIC: The Atherosclerosis Risk in Communities Study is carried out as a collaborative study supported by National Heart, Lung, and Blood Institute contracts (HHSN268201100005C, HHSN268201100006C, HHSN268201100007C, HHSN268201100008C, HHSN268201100009C, HHSN268201100010C, HHSN268201100011C, and HHSN268201100012C), R01HL087641, R01HL59367 and R01HL086694; National Human Genome Research Institute contract U01HG004402; and National Institutes of Health contract HHSN268200625226C. The authors thank the staff and participants of the ARIC study for their important contributions. Infrastructure was partly supported by Grant Number UL1RR025005, a component of the National Institutes of Health and NIH Roadmap for Medical Research.

GERA: The Genetic Epidemiology Research on Adult Health and Aging study was supported by grant RC2 AG036607 from the National Institutes of Health, grants from the Robert Wood Johnson Foundation, the Ellison Medical Foundation, the Wayne and Gladys Valley Foundation and Kaiser Permanente. The authors thank the Kaiser Permanente Medical Care Plan, Northern California Region (KPNC) members who have generously agreed to participate in the Kaiser Permanente Research Program on Genes, Environment and Health (RPGEH).

UKB: This study has been conducted using UK Biobank resource under Application Number 12514. UK Biobank was established by the Wellcome Trust medical charity, Medical Research Council, Department of Health, Scottish Government and the Northwest Regional Development Agency. It has also had funding from the Welsh Assembly Government, British Heart Foundation and Diabetes UK.

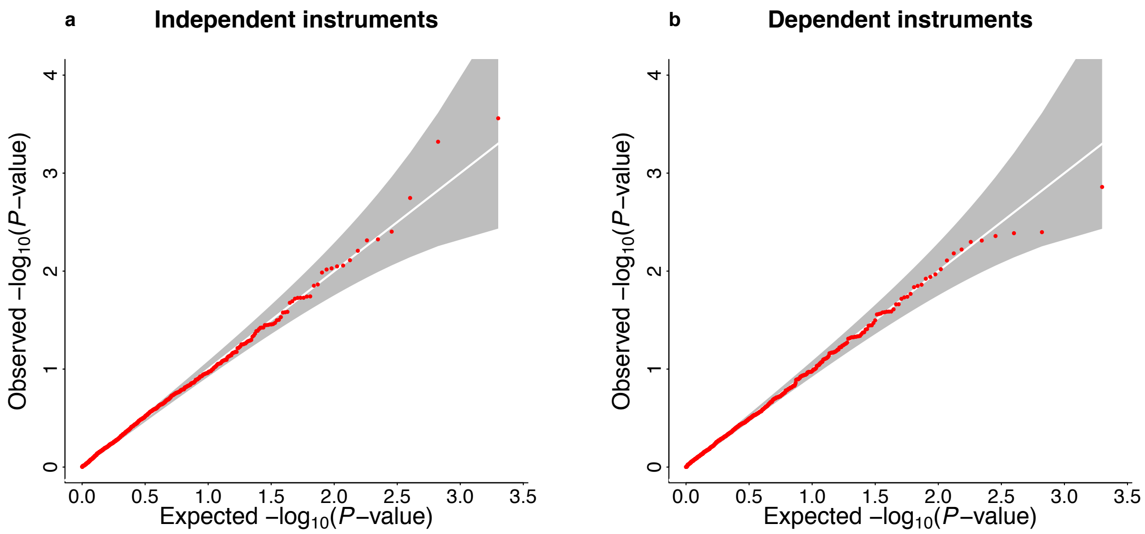

Supplementary Figure 1 QQ-plot of P_{GSMR} in the absence of causality. Shown is the result from 1,000 simulations under a model that there is no causal association between the exposure and outcome. In panel (a), genotypes of 100 unlinked SNPs were simulated from a binomial distribution. In panel (b), genotypes of 100 SNPs in LD were from the UKB. Effect sizes of SNPs on the exposure were estimated from a sample of 50,000 individuals, effect sizes for the outcome were estimated from an independent sample of 20,000 individuals, and the correlations between instruments were estimated from a third sample of 5,000 individuals. Details of the simulation can be found in **Supplementary Note 1**.

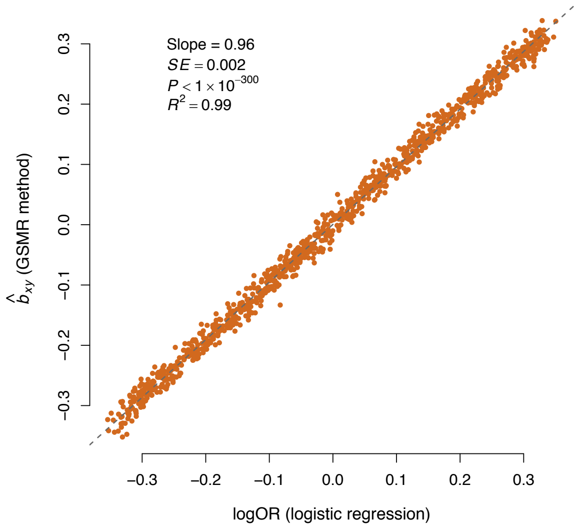

Supplementary Figure 2 Estimate of b_{xy} from GSMR vs. logOR from logistic regression. We described in the method section that b_{xy} is logOR of risk factor (x) on disease (y). We sought to confirm this by simulation (see **Supplementary Note 1** for details). We compared GSMR estimate of b_{xy} with logOR estimated from a logistic regression analysis of y_{01} (disease status on the observed scale) against x in one sample. The result shows that b_{xy} estimate is highly consistent with logOR, meaning that we can interpret exp(b_{xy}) as odds ratio. Note that in order to demonstrate the equivalence between b_{xy} and logOR we did not simulate a confounding factor in this case. In reality, if the association between x and y is partly confounded by unobserved confounding factors, the GSMR estimate of b_{xy} from two samples will be smaller than logOR estimated from logistic regression in one sample.

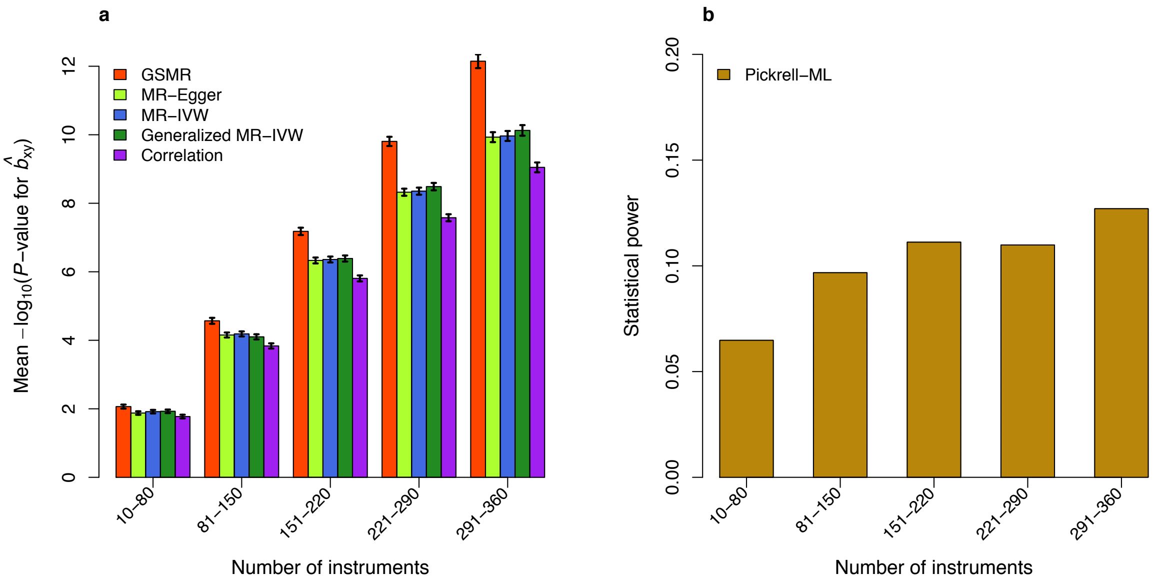

Supplementary Figure 3 Comparison of power between GSMR and five other methods using simulation. Details of the simulation are described in **Supplementary Note 1**. In brief, we simulated 1,000 independent genetic variants (z) that have effects on the outcome (y) mediated by the exposure (x). The variance in x explained by z (R^2_{zx}) was 0.60, and the variance in y explained by x (R^2_{xy}) was 0.005. We analyzed the simulated data using GSMR, Egger regression (MR-Egger)[1], the inverse-variance weighted method (MR-IVW)[2], the generalized linear regression (generalized MR-IVW)[3] and two methods used in Pickrell *et al.*[4] based on a random subset of the instruments, varying from 10 to all the genome-wide significant SNPs (P_{GWAS} < 5e−8) associated with x. We repeated the simulation 10,000 times. Shown in panel (a) is the mean – log10 based p-value in each bin (stratified by the number of instruments). Each error bar represents the 95% confidence interval of the mean estimate.

We focus on the simulation analysis based on independent SNPs because in practice we usually include in the GSMR analysis only the SNPs from a clumping analysis with a stringent LD r^2 threshold (e.g. 0.05), although GSMR accounts for remaining LD not removed by clumping (**Supplementary Figure 1b** and **Supplementary Table 1**). There are subtle differences (not statistically significant) which are due to the use of SNP correlations (all chance correlations because the SNPs are independent) computed from a reference sample for generalized MR-IVW.

Note that the two methods used in Pickrell *et al.* are not based on the MR framework. One method is to use the correlation in effect sizes of a set of top associated SNPs on exposure and outcome to assess the genetic overlap between x and y. Pickrell *et al.* developed another method that exploits the asymmetry of correlation (assuming that the correlation computed from SNPs associated with the exposure is different from that computed from SNPs associated with the

outcome) to infer causality by a maximum likelihood approach (referred to as Pickrell-ML). In panel (b), the statistical power is defined as the proportion of simulations in which the relative likelihood of the best non-causal model compared to the best causal model is smaller than 0.05. Because the hypothesis tested in the Pickrell-ML method is very different hypothesis from those in the other methods, the simulation result above needs to be interpreted with great caution.

It is also of note that we did not include the bivariate LD score regression (LDSC) method[18] in the comparison because 1) the LDSC method aims to estimate the genetic correlation between two phenotypes rather than the directional effect of one phenotype on another; 2) it assumes a polygenic model for the traits and utilizes variability in LD scores across SNPs to estimate the genetic variance and covariance, and therefore has only been applied to all genome-wide SNPs.

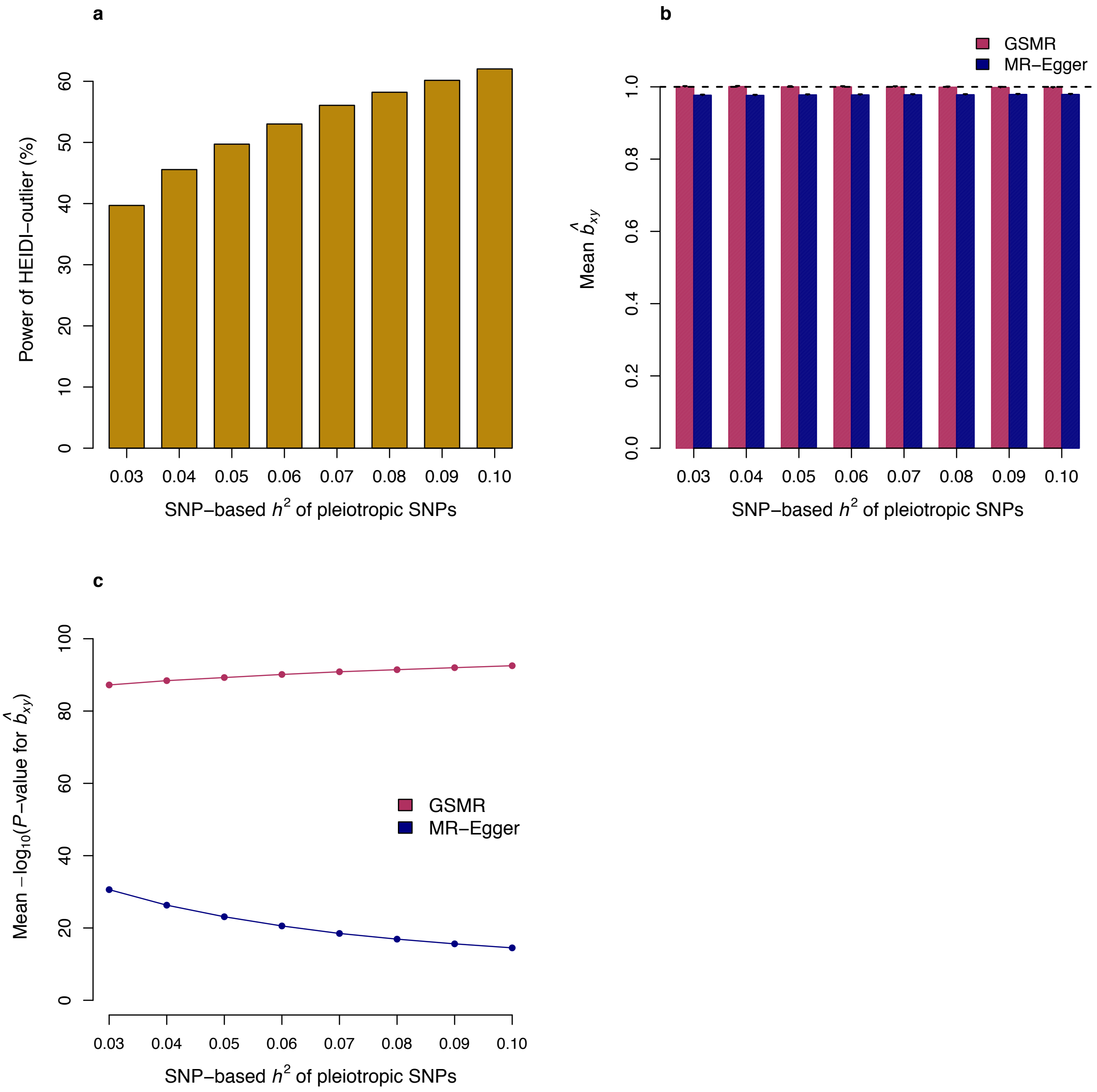

Supplementary Figure 4 Power of the HEIDI-outlier test and the (un)biasedness of $\hat{b}_{xy}$ in the presence of pleiotropy. Details of the simulation can be found in **Supplementary Note 1**. In brief, we simulated 500 SNPs (z_c) that have effects on the outcome (y) mediated by the exposure (x), and additional 50 SNPs (z_p) that have pleiotropic effects on both x and y. The variance in x explained by z ($R^2_{z_c x}$) is 0.4. The variance in x explained by z_p varies from 0.03 to 0.1 with an increment of 0.01. The variance in y explained by x (R^2_{xy}) is 0.05. Shown in the panel (a) is the power of HEIDI-outlier to detect pleiotropic instruments. Shown in the panel (b) is the mean estimate of b_{xy} from GSMR (after HEIDI-filtering) and that from MR-Egger across 1,000 replicates. Error bar represents the standard error of the mean. Panel (c) shows the mean $-\log_{10}$(P-value) for $\hat{b}_{xy}$ from the two methods.

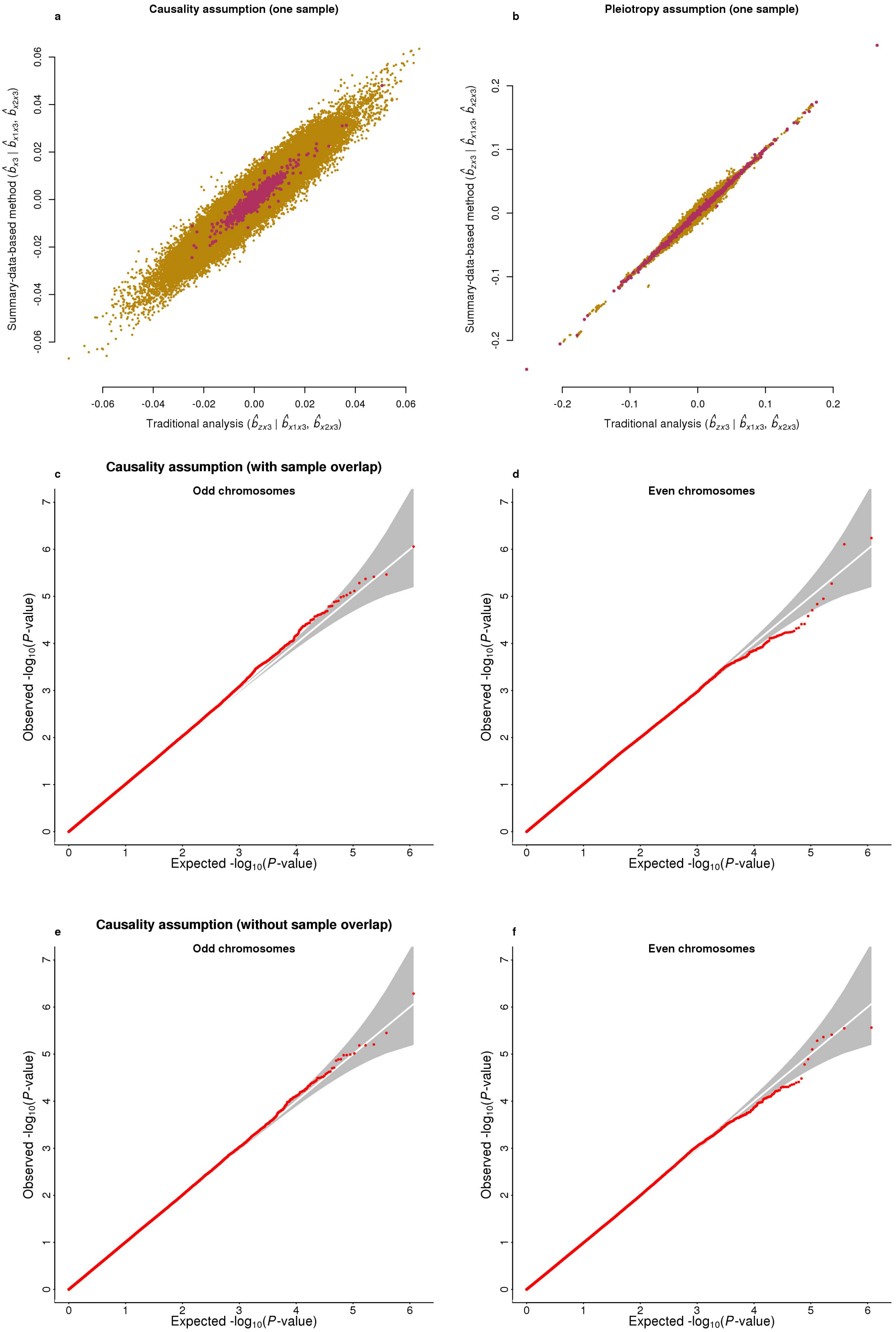
a
Causality assumption (one sample)
Summary-data-based method ($\hat{b}_{x3}$ | $\hat{b}_{x1x3}$, $\hat{b}_{x2x3}$)
Traditional analysis ($\hat{b}_{zx3}$ | $\hat{b}_{x1x3}$, $\hat{b}_{x2x3}$)
b
Pleiotropy assumption (one sample)
Summary-data-based method ($\hat{b}_{zx3}$ | $\hat{b}_{x1x3}$, $\hat{b}_{x2x3}$)
Traditional analysis ($\hat{b}_{zx3}$ | $\hat{b}_{x1x3}$, $\hat{b}_{x2x3}$)
Causality assumption (with sample overlap)
c
Odd chromosomes
d
Even chromosomes
Causality assumption (without sample overlap)
e
Odd chromosomes
f
Even chromosomes
Observed -log$_{10}$(P-value)
Expected -log$_{10}$(P-value)

Supplementary Figure 5 Comparison of mtCOJO with the exact approach (i.e. linear regression). We conducted simulations in the UKB data where we simulated 3 correlated traits (x_1, x_2 and x_3) based on the real genotype data (details of the simulation method can be found in **Supplementary Note 2**). Shown in panels (a) and (b) are comparisons of the estimates of SNPs effects on x_3 conditional on the genetic values of x_1 and x_2 (i.e. g_{x1} and g_{x2}) in one sample under two different models. The simulated causal variants are highlighted in red. Shown in panels (c) and (d) are QQ plots of mtCOJO p-values for $\hat{b}_{zx_3}|(\hat{b}_{x_1x_3}, \hat{b}_{x_2x_3})$ when the samples for x_1 (n_1 = 30,000) and x_2 (n_2 = 20,000) are two non-overlapping subsets of that for x_3 (n_3 = 108,039). Shown in panel (e) and (f) are QQ plots of mtCOJO p-values when the samples for x_1 (n = 30,000), x_2 (n = 20,000) and x_3 (n = 50,000) are completely independent.

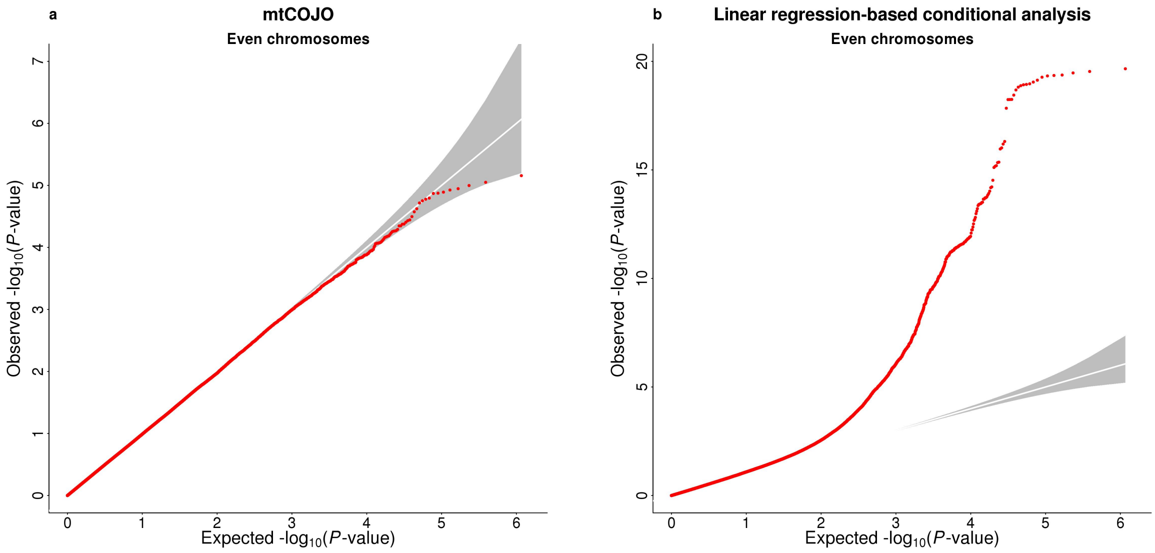

Supplementary Figure 6 QQ plots of the conditional p-values of SNPs from mtCOJO and linear regression-based conditional analysis. We performed a simulation to investigate whether the estimate of conditional SNP effect from mtCOJO is biased or not due to the shared genetic effect (details of the simulation can be found in **Supplementary Note 2**). Shown in panel (a) are the p-values for the SNP effects on the phenotype (y) conditional on x from the mtCOJO analysis using summary data. The p-values from the linear regression-based conditional analysis are shown in panel (b). In the simulation, the genetic variants (z) that only have effects on the covariate (x) are on the even chromosomes, and the genetic variants (z_c) that have confounding effects on both x and y are on the odd chromosomes.

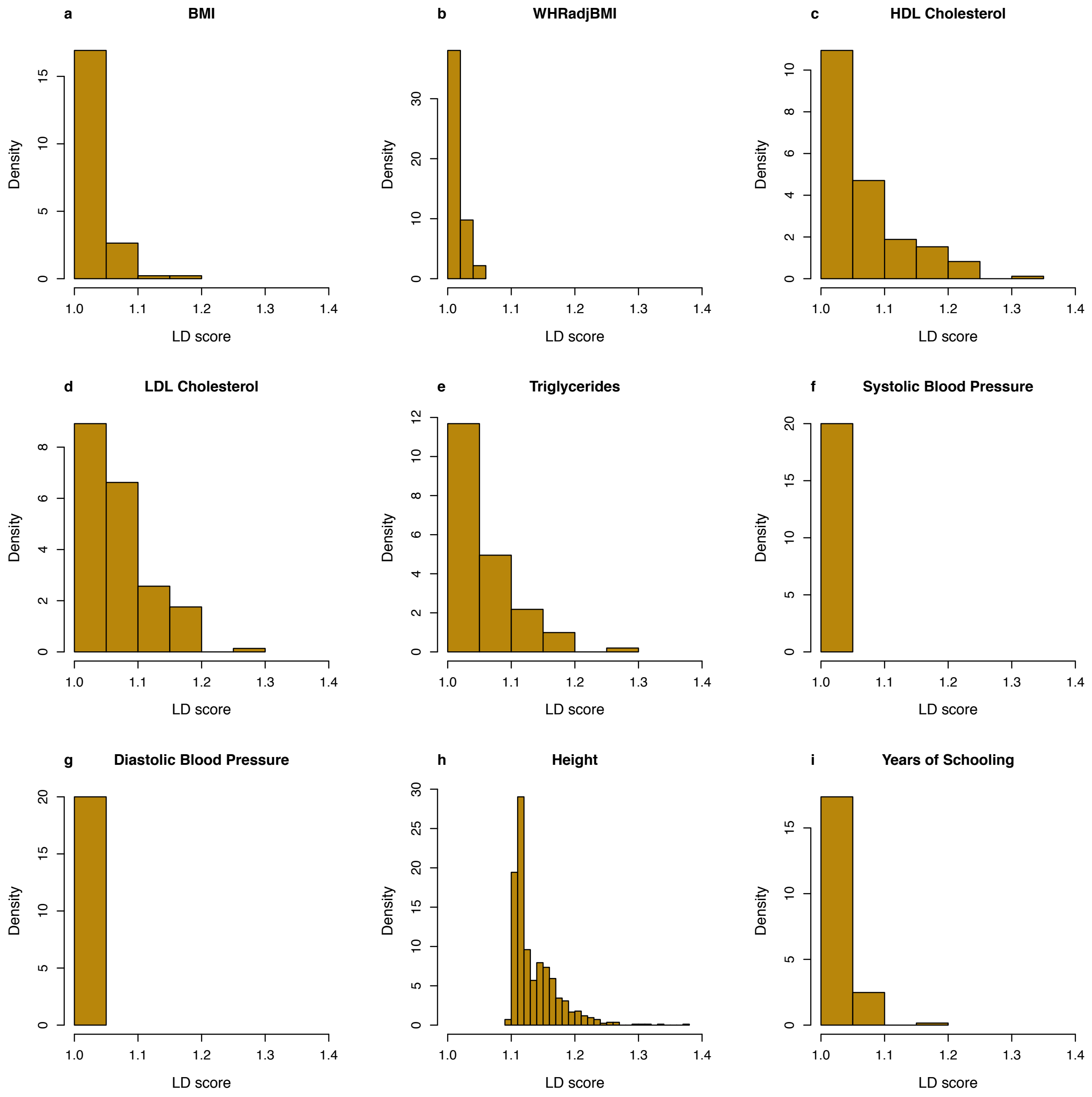

Supplementary Figure 7 Distribution of the LD scores of the instruments for each risk factor. The LD score of a SNP instrument is defined as the sum of LD r^2 between the target instrument and all instruments (including the target)[19].

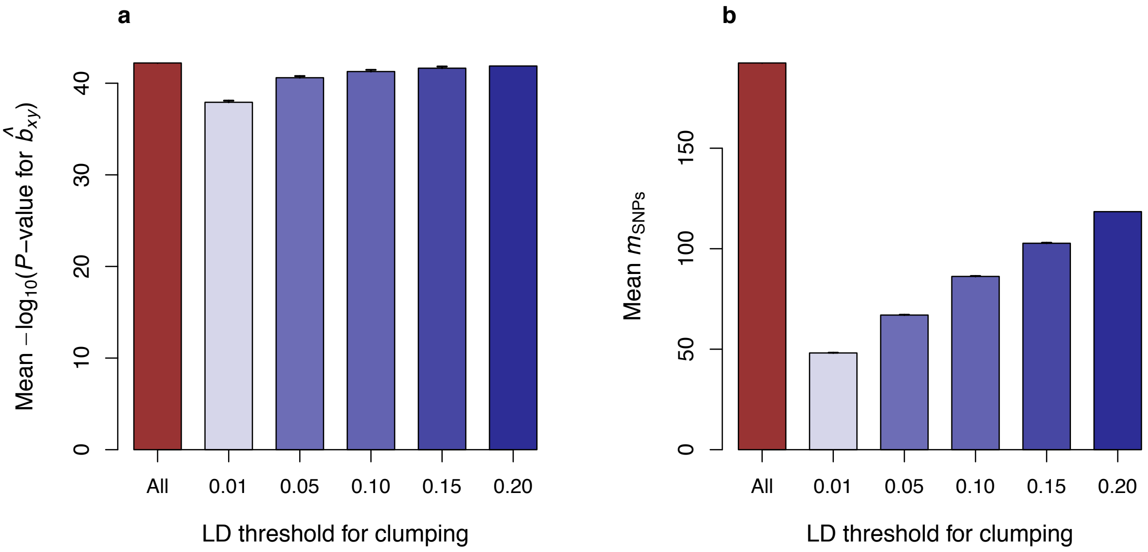

Supplementary Figure 8 Statistical power of GSMR at different LD thresholds for clumping. Details of the simulation are described in **Supplementary Note 1**. In brief, we extracted genotypes of SNPs of a 15Mb genomic segment on chromosome 22 from the UKB data. After LD pruning with a LD r^2 threshold of 0.5 and a window size of 100kb, 2,006 SNPs were retained. We then randomly sampled 100 SNPs as the causal variants for simulation under a causal model. To calibrate the extent to which the power of GSMR changes with LD among the instruments, we clumped the instruments at a LD threshold varying from 0.01 to 0.2. We repeated the simulation 1,000 times and reported the mean of $-\log_{10}(P_{\text{GSMR}})$ (panel a) and the mean number of instruments (panel b) at each clumping threshold. Error bars represent standard errors.

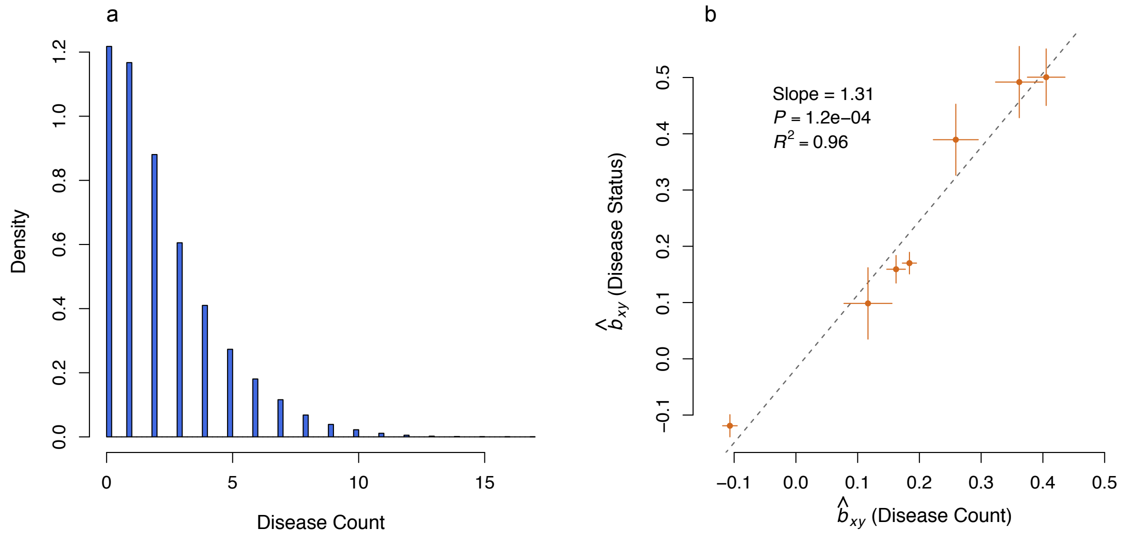

Supplementary Figure 9 Estimates of b_{xy} of 7 modifiable risk factors on 'Disease Status' vs. those on 'Disease Count'. 'Diseased Status' (DS) is a dichotomous trait to describe whether an individual is affected by any of the 22 diseases or not in the GERA and UKB data (see **Supplementary Table 4** for a list of the diseases). 'Disease Count' (DC) is a categorical trait to count the number of diseases that affect an individual. Both traits can be used to measure the general health status of an individual. Shown in panel (a) is the distribution of DC in the combined GERA and UKB data. The phenotypic correlation between DC and DS is 0.57 in the combined data. Shown in panel (b) is a comparison between the GSMR estimates for the two phenotypes. Error bars represent standard errors. The dashed line represents the regression slope of regressing $\hat{b}_{xy}$ for DS on $\hat{b}_{xy}$ for DC. The result shows that $\hat{b}_{xy}$ for DS is highly consistent with that for DC while the standard error for the former is larger than that for the latter, suggesting that b_{xy} for DC approximately equals that for DS and that it is more powerful to detect the causative effect on a risk factor on general health status using DC than using DS.

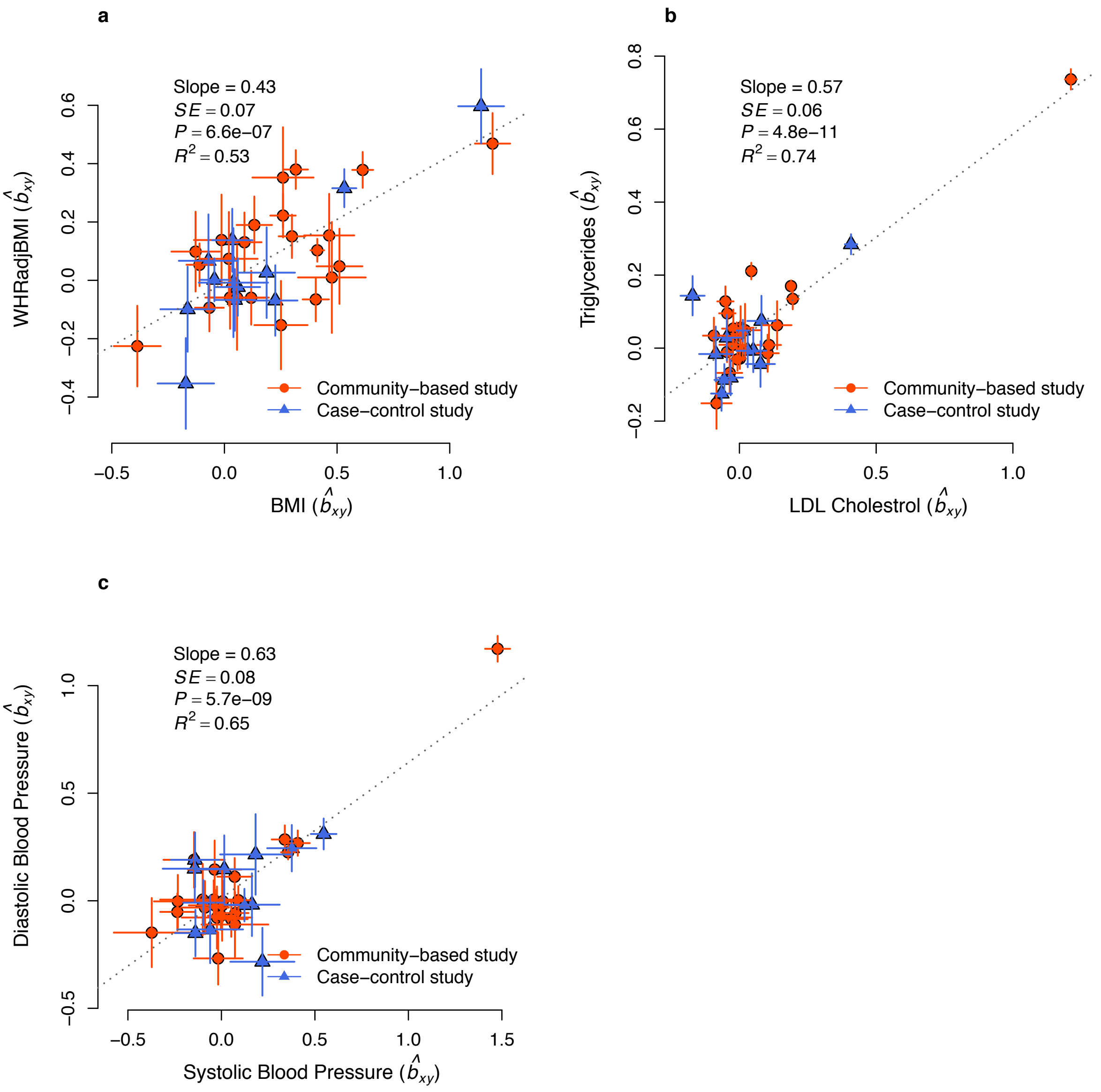

Supplementary Figure 10 Comparisons of $\hat{b}_{xy}$ between correlated risk factors. Shown are comparisons of $\hat{b}_{xy}$ (a) between BMI and WHRadjBMI, (b) between LDL-c and TG, and (c) between SBP and DBP. Error bars represent standard errors. Dashed lines represent the regression slopes.

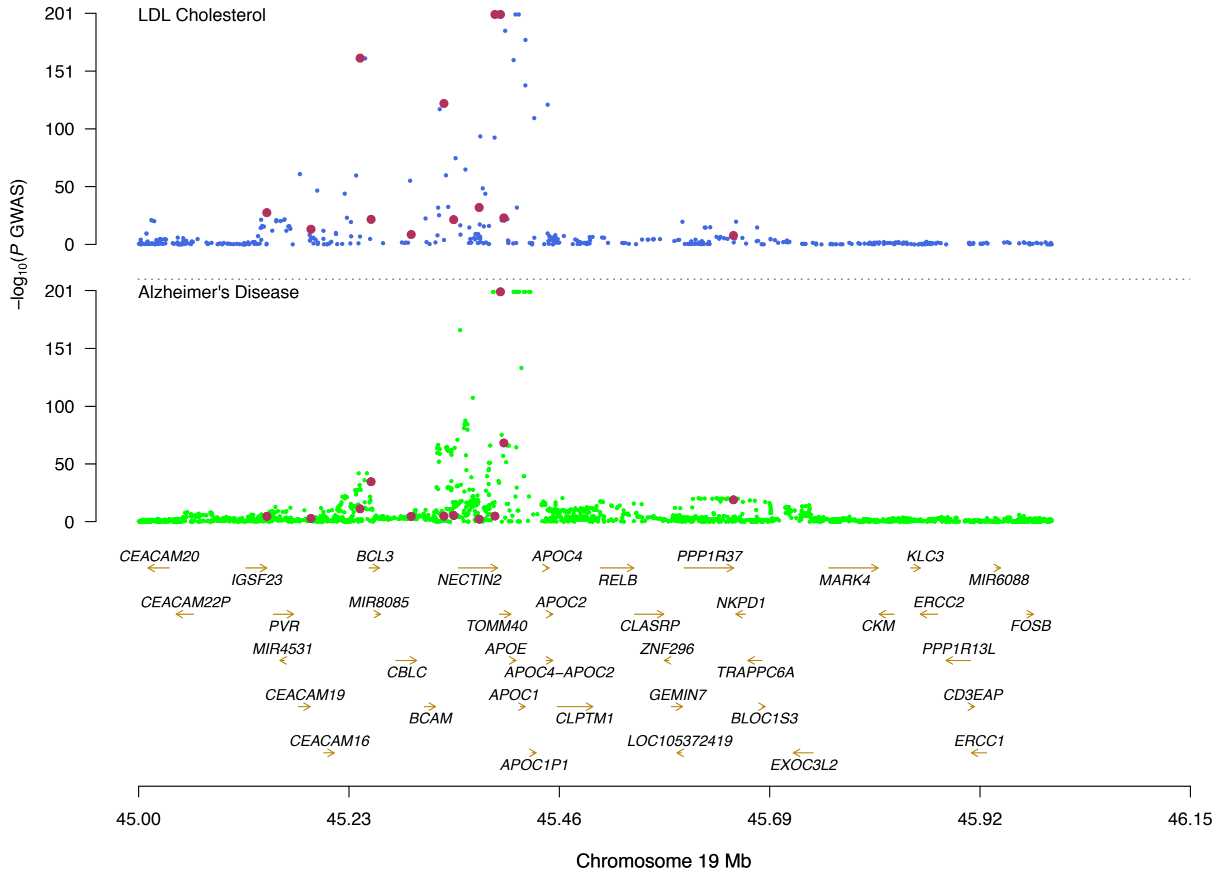

Supplementary Figure 11 Regional GWAS plot for LDL-c and AD at the *APOE* locus. Highlighted are the near-independent GWS SNPs removed by the HEIDI-outlier. The p-values are truncated at 1e−200.

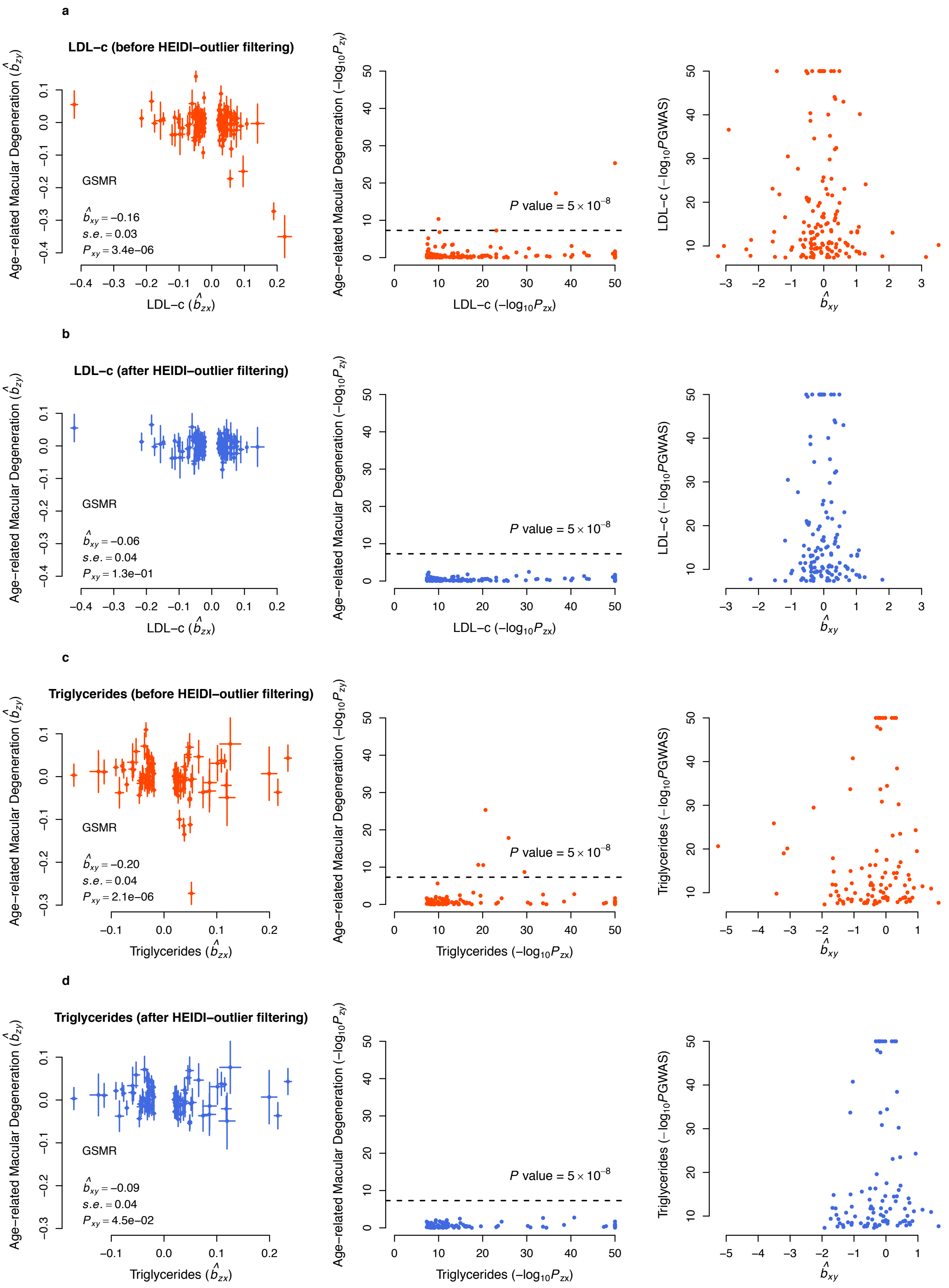

Supplementary Figure 12 GSMR analyses to test for the effects of LDL-c and TG (TG) on AMD (AMD) with and without HEIDI-outlier filtering. The SNP instruments were ascertained based on the GWAS p-values for LDL-c in panels (a) and (b), and TG in panels (c) and (d). Shown in the first column are the plots of SNP effect sizes for AMD against those for LDL-c (or TG), with the

corresponding GWAS p-value plots shown in the second column. Shown in the last column are the GWAS p-values for LDL-c (or TG) against the estimates of b_{xy}. In panels (b) and (d), SNPs detected as pleiotropic outliers by the HEIDI-outlier approach were removed. The error bars in the column represent the standard errors. The dashed lines on the plots in the second column represent the GWAS threshold p-value of 5×10^{-8}. P-values were truncated at 1.0×10^{-50} for better graphic presentation.

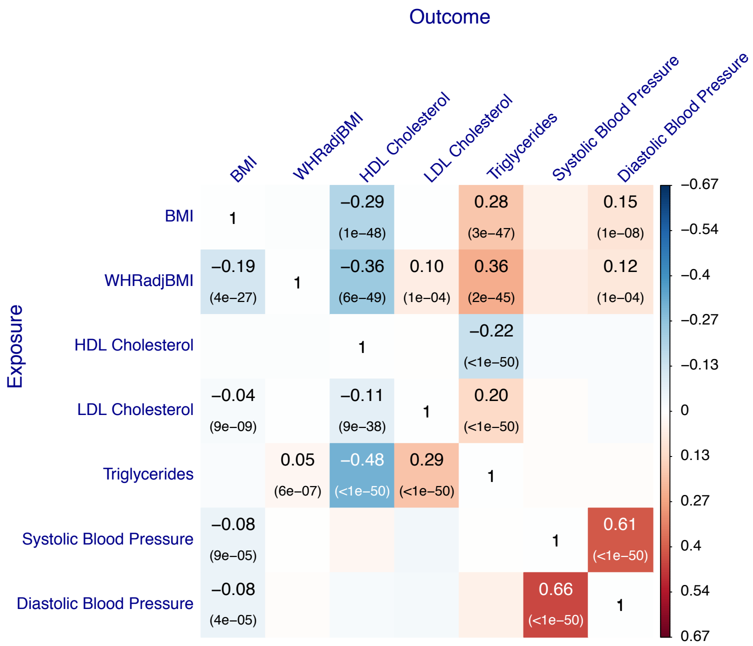

Supplementary Figure 13 Causative associations between each pair of modifiable risk factors. Colors represent the effect sizes (b_{xy}) of a risk factor (row-wise) on another risk factor (column-wise), red for positive effects and blue for negative effects. Effect sizes and p-values (in the brackets) are labeled on the associations with p-value < 1.2×10^{-3}).

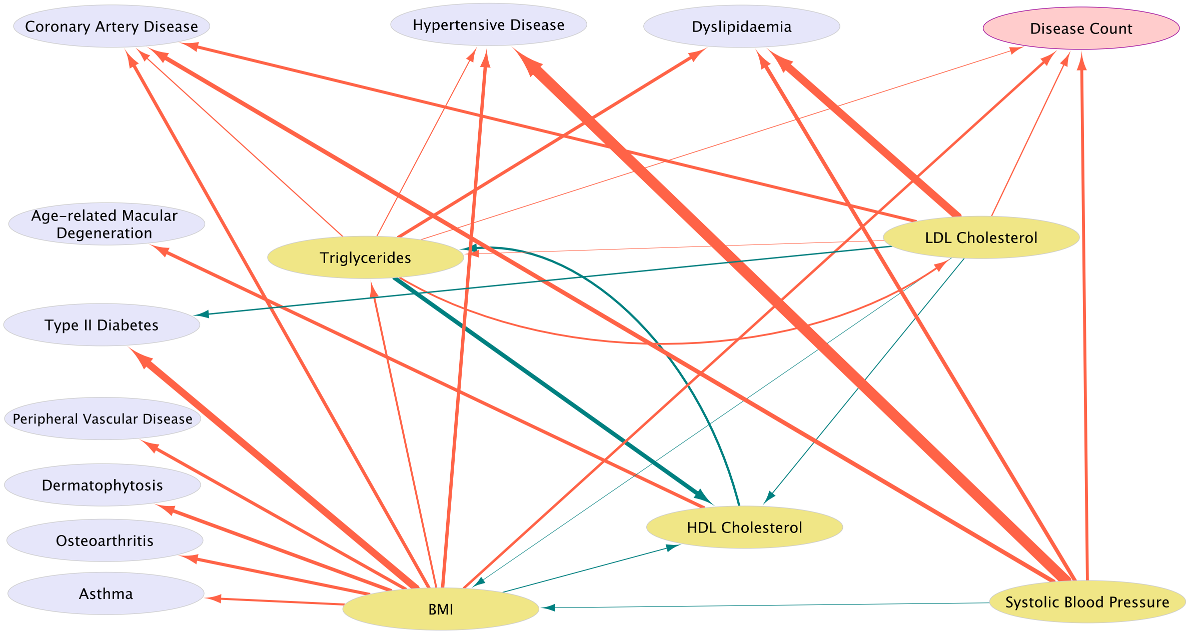

Supplementary Figure 14 A network of causative associations between modifiable risk factors and common diseases identified from the conditional GSMR analysis. Red line represents the positive effect (risk effect for risk factor -> disease), and green line represents the negative effect (protective effect for risk factor -> disease). The width of a line represents the absolute value of $\hat{b}_{xy}$ (logOR for risk factor -> disease).

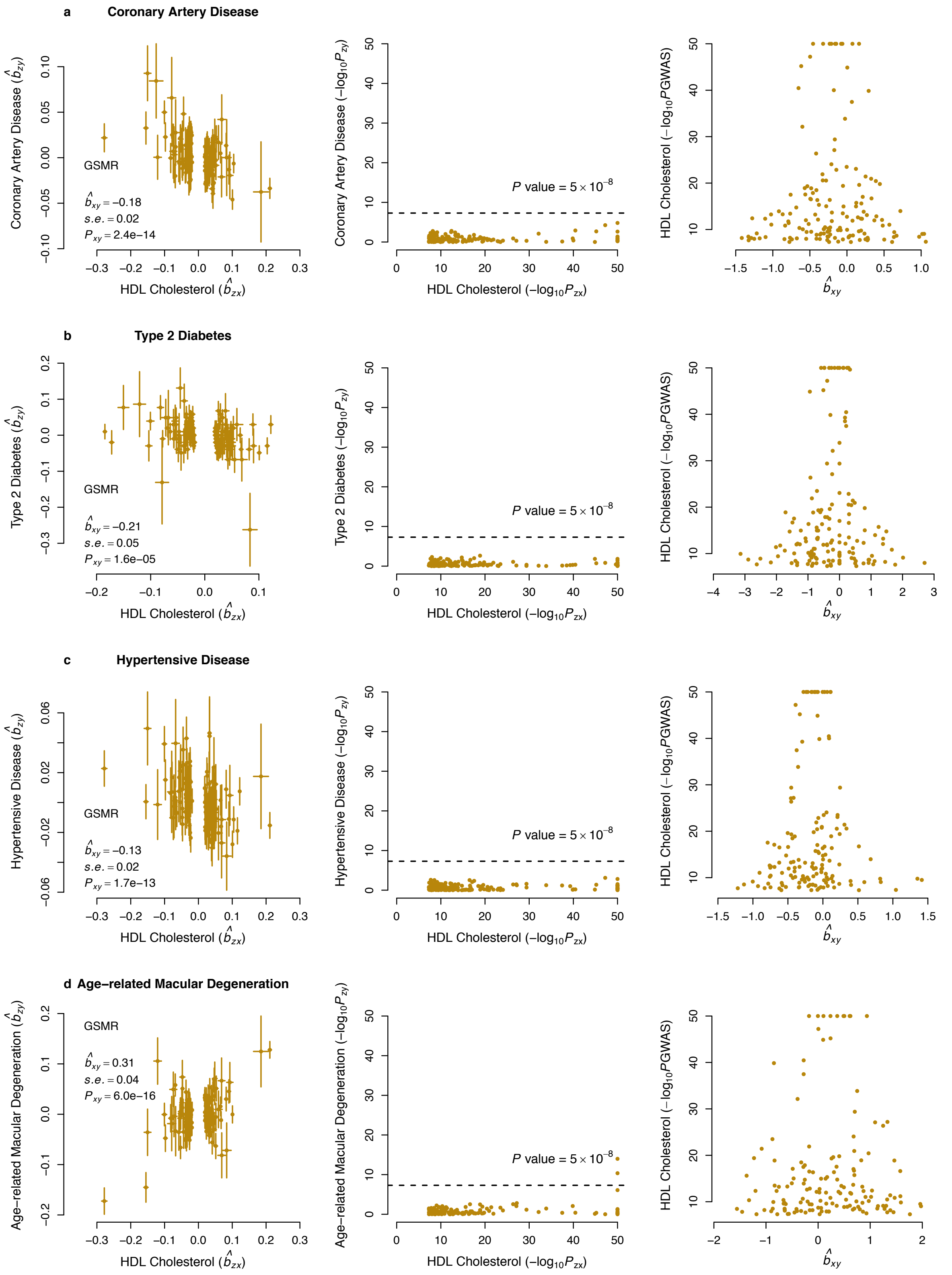

Supplementary Figure 15 The effects of HDL-c on CAD, T2D, hypertension and AMD. The GSMR analyses were performed with HEIDI-outlier filtering. The SNP instruments were ascertained based on the GWAS p-values for HDL-c. Shown in the first column are the plots of SNP effect sizes for disease against those for HDL-c, with the corresponding GWAS p-value plots shown in

the second column. Shown in the last column are the GWAS p-values for HDL-c against the estimates of b_{xy}. The error bars in the first column represent the standard errors. The dashed lines in the second column represent the GWAS threshold p-value of 5×10^{-8}. P-values were truncated at 1.0×10^{-50} for better graphic presentation.

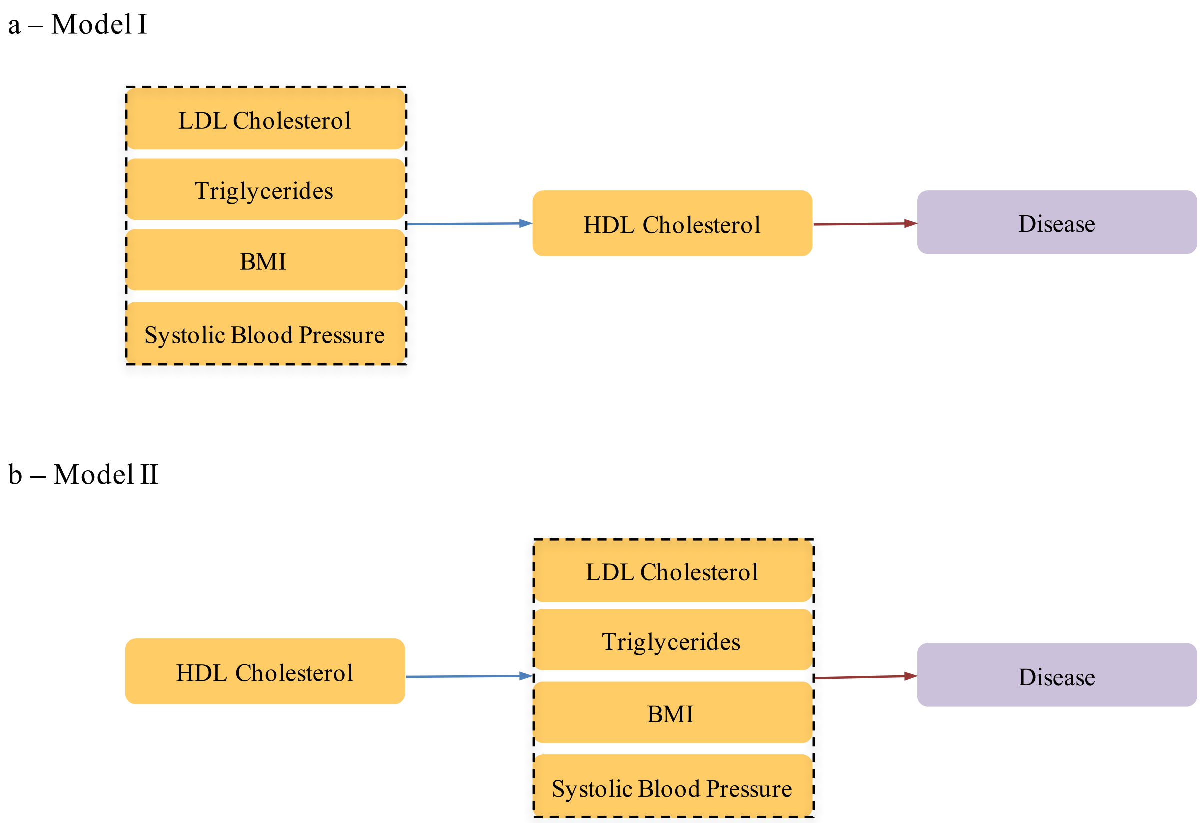

Supplementary Figure 16 Two hypothetical models for the effect of HDL-c on a disease. In model I, the effect of HDL-c on the disease is a consequence of other risk factors (i.e. HDL-c is a mediating factor). In model II, the effect of HDL-c on the disease is mediated by other risk factors (i.e. HDL-c is a driving factor).

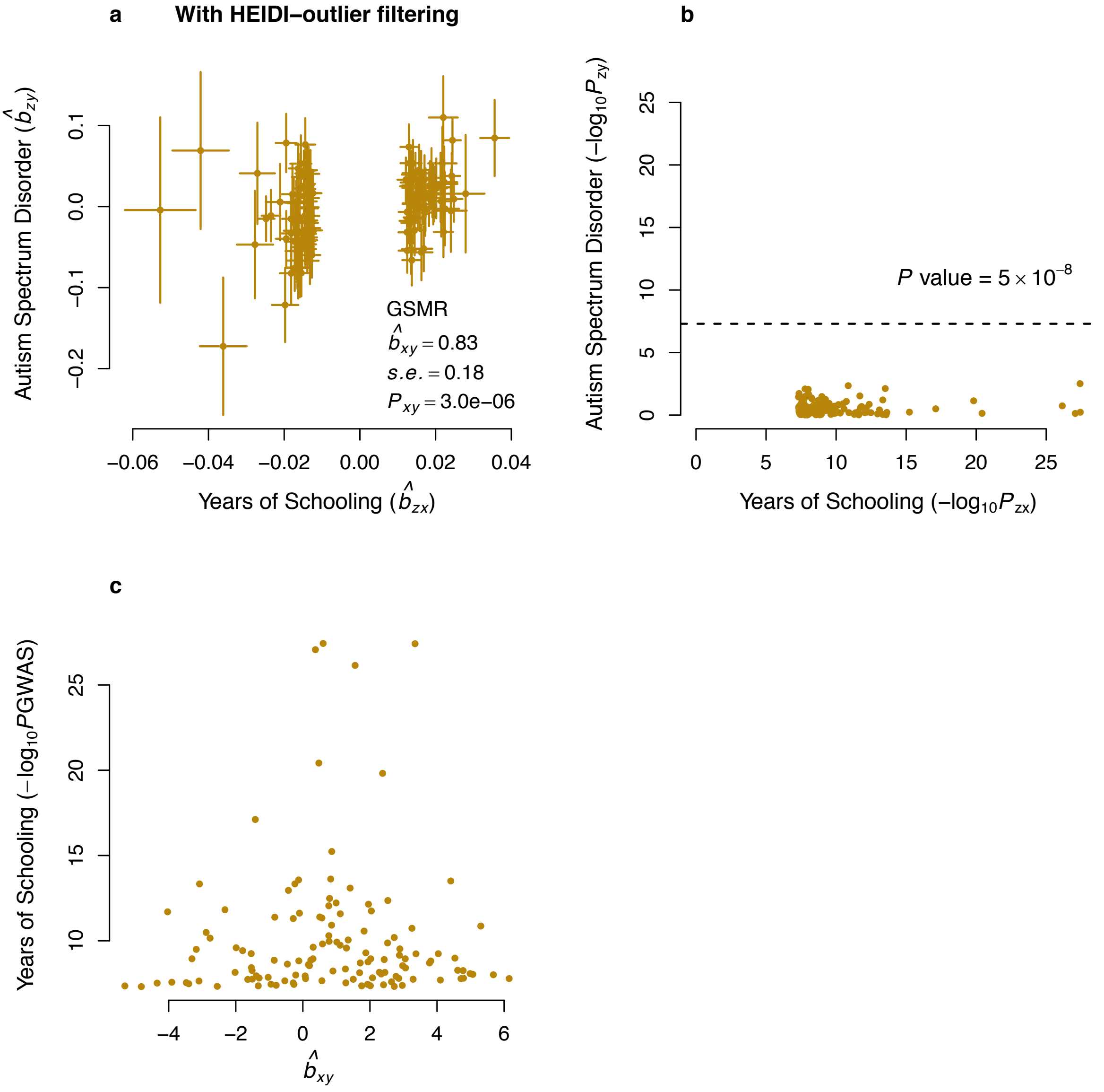

Supplementary Figure 17 GSMR analysis to test for the effect of EduYears on ASD with HEIDI-outlier filtering. The SNP instruments were ascertained based on the GWAS p-values for EduYears. Shown in panel (a) is the plot of SNP effect sizes for ASD against those for EduYears, with the corresponding GWAS p-value plots shown in panel (b). Shown in panel (c) are the GWAS p-values for EduYears against the estimates of b_{xy}. The error bars in the panel (a) represent the standard errors. The dashed line in the panel (b) represents the GWAS threshold p-value of $5{\times}10^{-8}$. *P*-values were truncated at $1.0{\times}10^{-50}$.

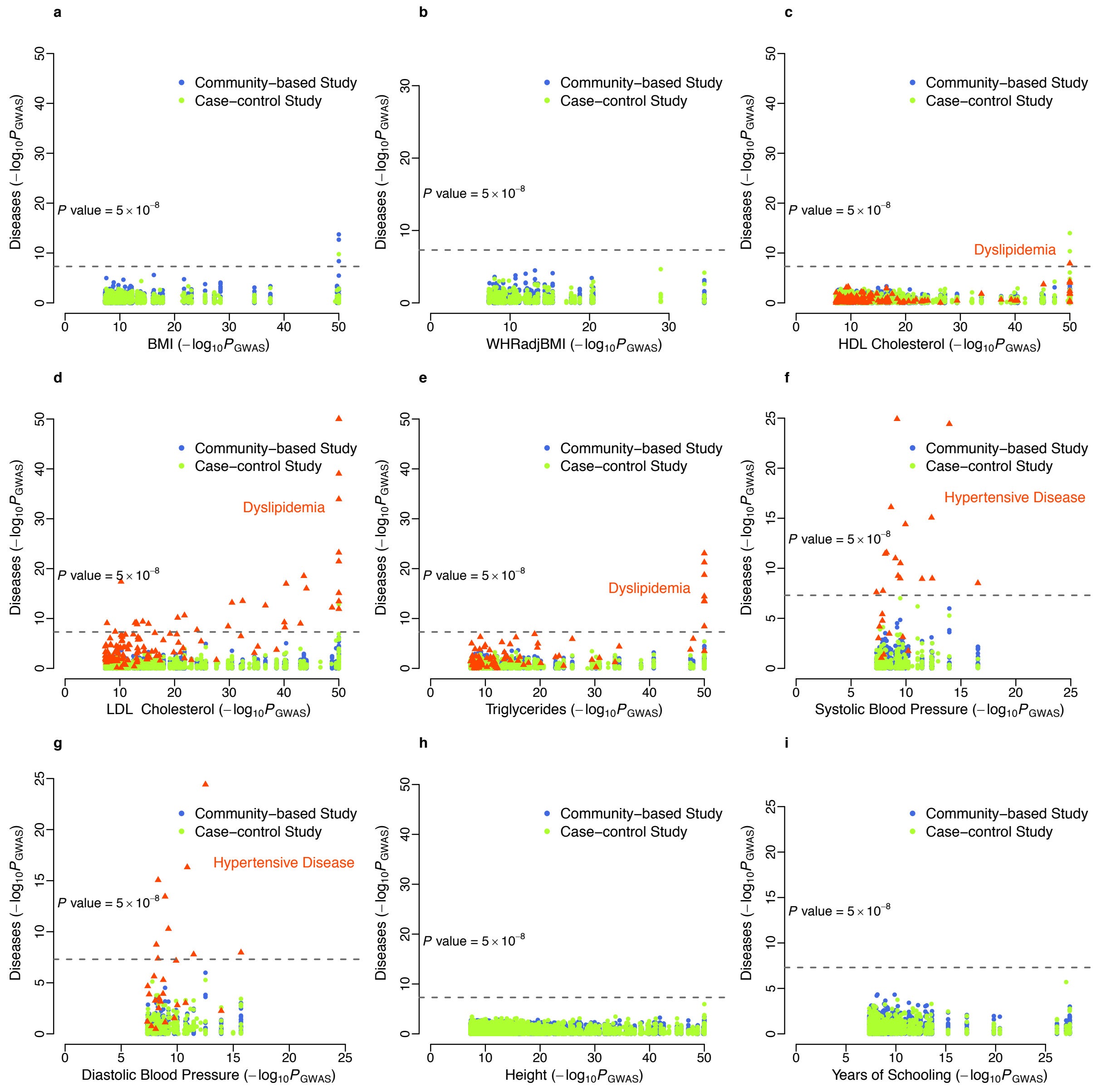

Supplementary Figure 18 Association p-values of the SNP instruments with risk factors vs. those with diseases. The dashed horizontal line represents the GWAS p-value threshold of $5{\times}10^{-8}$. We highlighted the p-values of SNPs for dyslipidemia in panels (c), (d) and (e), and those for hypertensive disease in panels (f) and (g). The coordinates of panels (a), (c), (d), (e) and (h) are truncated at 50.

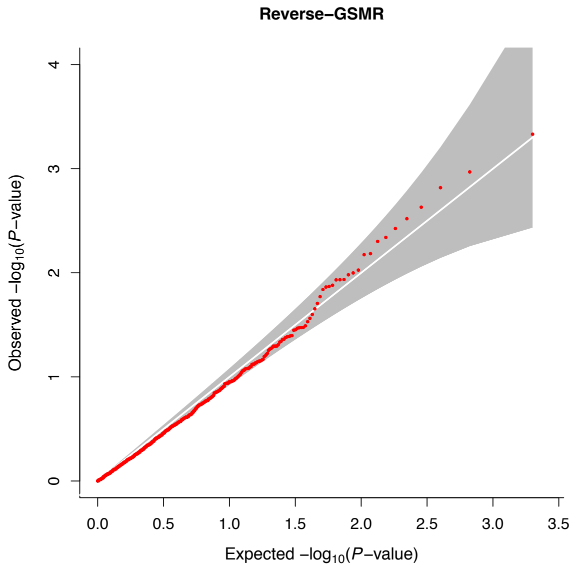

Supplementary Figure 19 QQ-plot of $P_{\text{reserve-GSMR}}$ under the null hypothesis. The result is from simulation under the null that there is a forward effect but no reverse effect. Details of the simulations can be found in **Supplementary Note 1**.

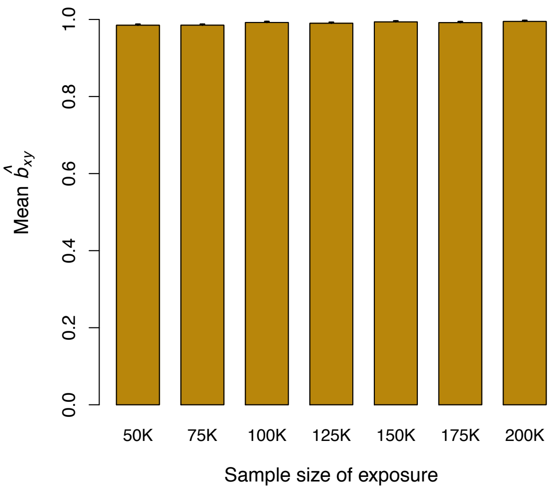

Supplementary Figure 20 Estimate of b_{xy} as a function of sample size. The results are from simulation based on 100 unlinked SNPs with different sample sizes for the exposure. The bar represents the standard error of mean $\hat{b}_{xy}$. Details of the simulations can be found in **Supplementary Note 1**.

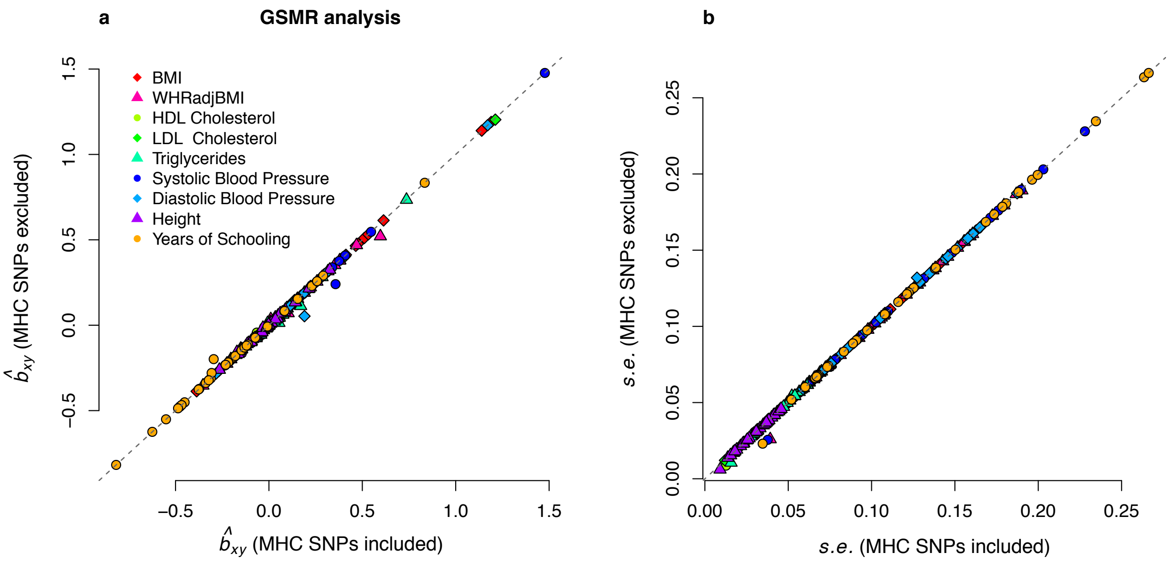

Supplementary Figure 21 Estimates of b_{xy} from GSMR analyses excluding SNPs in the MHC region. To test if the GSMR estimates would be biased by SNPs in the MHC regions, we excluded the SNPs in the MHC locus[20] (chr6:2,957,005-33,377,699, hg19) before the clumping analysis. Shown in panel (a) is a comparison between the $\hat{b}_{xy}$ estimates from GSMR with and without MHC SNPs. Shown in panel (b) is a comparison of the corresponding standard errors. The dashed lines are diagonal lines with intercept 0 and slope 1.

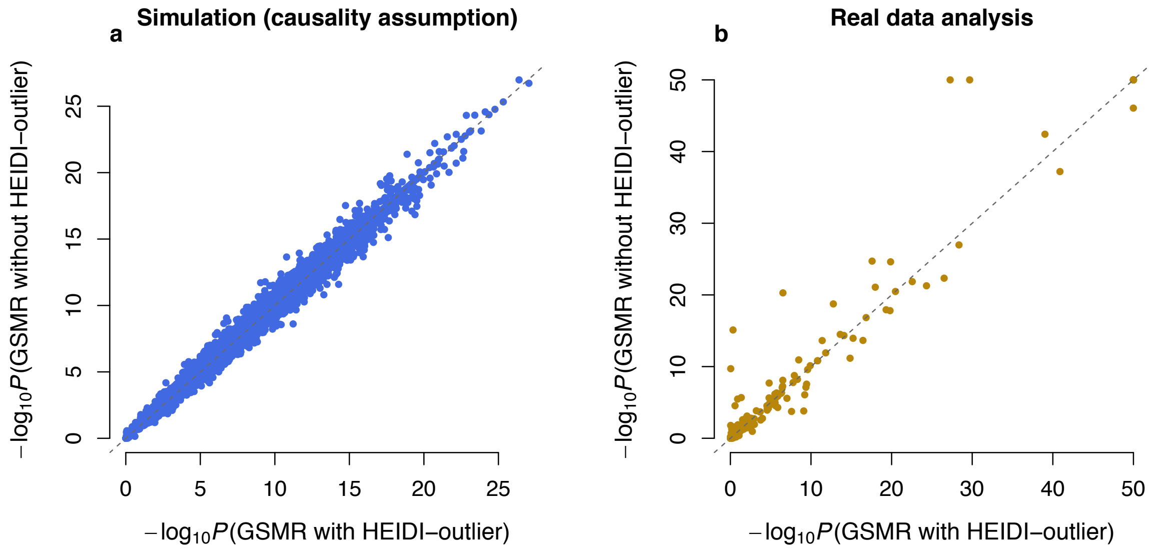

Supplementary Figure 22 Comparison of power between GSMR with and without HEIDI-outlier filtering. Shown in the plot are the comparisons by simulation (a) and real data analysis (b). The simulation method is described in **Supplementary Note 1**. In brief, we simulated 1,000 genetic variants (z) that all have causal effects on outcome (y) mediated by exposure (x). The variance in x explained by z (R^2_{zx}) was 0.60, and the variance in y explained by x (R^2_{xy}) was 0.005. We conducted GSMR analyses with and without HEIDI-outlier filtering, using a random subset of the genetic instruments, varying from 10 to all the genome-wide significant SNPs (P_{GWAS} < 5e−8) associated with x. We repeated the simulation 10,000 times. Shown in the panel (a) is the p-value for test significance of $\hat{b}_{xy}$ in the simulation. Shown in the panel (b) is the p-value of $\hat{b}_{xy}$ for all the associations in the real data analyses. The dashed lines are diagonal lines with intercept 0 and slope 1. For better graphic presentation, the coordinates in panel (b) are truncated at 50. The mean –log10 of p-values are 6.80 and 6.80 for analyses with and without HEIDI-outlier filtering in simulation, and are 5.88 and 6.77 in real data analyses.

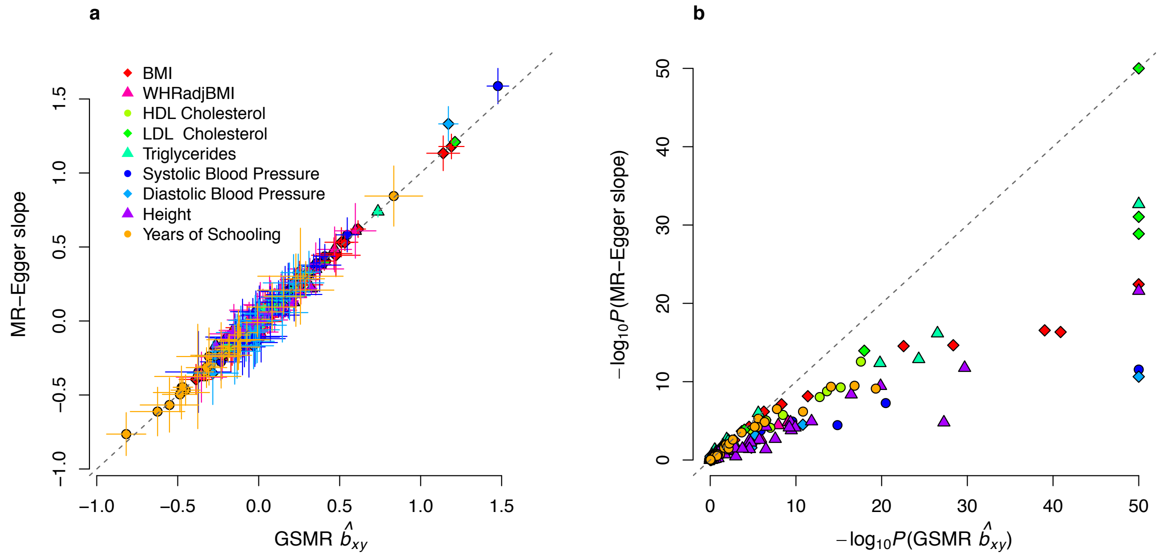

Supplementary Figure 23 Comparison between GSMR and Egger regression. Shown are the results from the analyses of all the risk factors and all the diseases included in this study. Error bars represent standard errors and the dashed lines are diagonal lines with intercept 0 and slope 1. Egger regression is a MR analysis approach that estimates and tests the mediation effect by regressing $\hat{b}_{zy}$ (the effect size of the instrument on the disease) on $\hat{b}_{zx}$ (the effect size of the instrument on the risk factor)[1]. Shown in panel (a) is a plot of GSMR estimate of b_{xy} (with HEIDI-filtering) against the MR-Egger estimate of b_{xy}, suggesting that the estimates from these two approaches are almost identical. Shown in panel (b) is the plot of the p-value to test for the significance of $\hat{b}_{xy}$ from GSMR against that from MR-Egger (for better graphic presentation, the coordinates are truncated at 50), suggesting that in general GSMR is more powerful than MR-Egger. This is further confirmed by simulations (**Supplementary Fig. 3**).

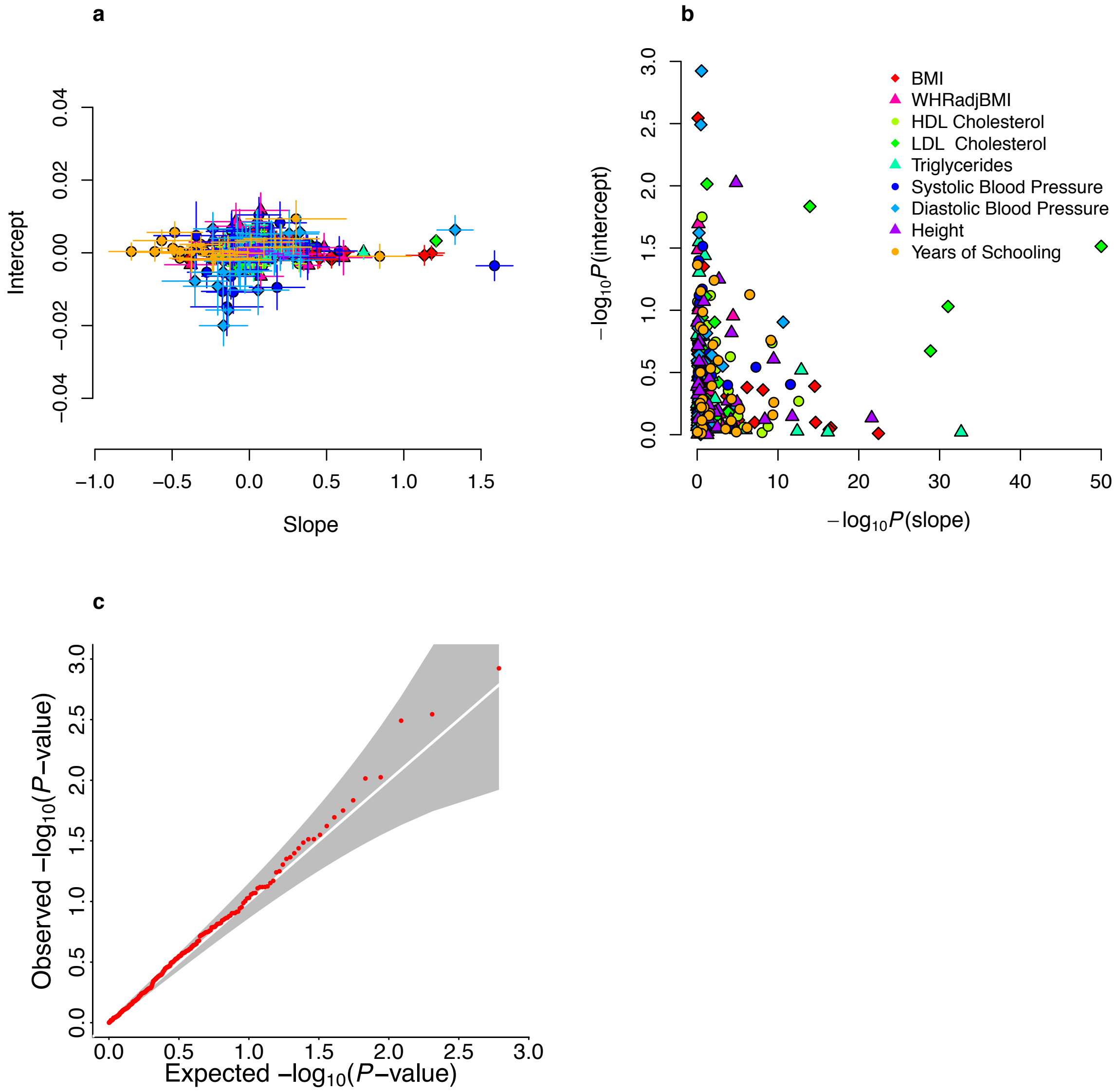

Supplementary Figure 24 Estimates of intercept vs. slope from MR-Egger. Shown are the results from the analyses of all the risk factor and all the diseases included in this study, with pleiotropic outliers removed by HEIDI-outlier. In MR-Egger, the intercept is used to test for evidence of type-II pleiotropy and the slope is used to test for causality[1]. Panel (a) shows the estimate of intercept ($\hat{b}_0$) from MR-Egger compared with the estimate of slope ($\hat{b}_{xy}$) from MR-Egger. There is no correlation between intercept and slope. Error bars represent standard errors. Panel (b) shows the significance test p-value (from regression analysis) for the intercept compared with the p-value for the slope. These results show that there is little evidence of type-II pleiotropy once the pleiotropic outliers have been removed. Panel (c) is a QQ-plot of the p-values to test the significance of $\hat{b}_0$.

Supplementary Table 1 GSMR estimate of b_{xy} from simulation under a causal model

**Mean b_{xy} (s.e.m.)**	**GSMR**						**Generalized MR-IVW**		
	**SNP instruments are independent**			**SNP instruments are in LD**			**SNP instruments are in LD**		
	$R_{xy}^2 = 0$	$R_{xy}^2 = 0.05$	$R_{xy}^2 = 0.1$	$R_{xy}^2 = 0$	$R_{xy}^2 = 0.05$	$R_{xy}^2 = 0.1$	$R_{xy}^2 = 0$	$R_{xy}^2 = 0.05$	$R_{xy}^2 = 0.1$
$R_{zx}^2 = 0.15$	0.000 (0.0004)	0.987 (0.003)	0.988 (0.002)	-0.001 (0.0006)	0.988 (0.003)	0.994 (0.002)	-0.001 (0.0006)	0.995 (0.003)	1.000 (0.002)
$R_{zx}^2 = 0.2$	0.000 (0.0003)	0.993 (0.002)	0.993 (0.002)	0.000 (0.0005)	0.997 (0.002)	0.996 (0.002)	0.000 (0.0006)	1.002 (0.002)	1.000 (0.002)
$R_{zx}^2 = 0.3$	0.000 (0.0003)	0.994 (0.002)	0.995 (0.001)	0.000 (0.0004)	0.998 (0.002)	1.001 (0.001)	0.000 (0.0003)	1.000 (0.002)	1.001 (0.001)

Note: See **Supplementary Note 1** for details about the simulations. Generalized MR-IVW represents the weighted generalized linear regression[3]. Shown are the mean estimate and standard error of the mean estimate (s.e.m.) from 1,000 simulation replicates.

Supplementary Table 2 GSMR estimate of b_{xy} from simulation under a pleiotropic model

**Mean b_{xy} (s.e.m.)**	$R^2_{zp} = 0.03$	$R^2_{zp} = 0.04$	$R^2_{zp} = 0.05$	$R^2_{zp} = 0.06$	$R^2_{zp} = 0.07$	$R^2_{zp} = 0.08$	$R^2_{zp} = 0.09$	$R^2_{zp} = 0.10$
SNP instruments are independent	0.000 (0.0004)	0.000 (0.0004)	0.000 (0.0004)	0.000 (0.0004)	0.000 (0.0004)	0.000 (0.0005)	0.000 (0.0005)	0.000 (0.0005)
SNP instruments are in LD	0.005 (0.007)	-0.006 (0.007)	-0.001 (0.007)	-0.006 (0.007)	-0.007 (0.007)	0.002 (0.007)	0.002 (0.007)	0.001 (0.007)

Note: Details of the simulation are described in **Supplementary Note 1**. We set b_{xy} = 0 to investigate the (un)biasedness of the estimate of b_{xy} under a pleotropic model. R^2_{zp} is the proportion of variance in the exposure explained by all the SNPs. Shown are the mean and standard error of the mean (s.e.m.) of an estimate from 1,000 simulation replicates.

Supplementary Table 3 Descriptive summary of GWAS summary data for the 9 risk factors

Risk factor	*n*	Publication	URL
BMI	322,154	Locke *et al*. 2015 Nature	https://www.broadinstitute.org/collaboration/giant/index.php/GIANT_consortium_data_files
WHRadjBMI	210,088	Shungin *et al*. 2015 Nature	https://www.broadinstitute.org/collaboration/giant/index.php/GIANT_consortium_data_files
HDL-c	188,577	GLGC 2013 Nature Genetics	http://csg.sph.umich.edu//abecasis/public/lipids2013/
LDL-c	188,577	GLGC 2013 Nature Genetics	http://csg.sph.umich.edu//abecasis/public/lipids2013/
TG	188,577	GLGC 2013 Nature Genetics	http://csg.sph.umich.edu//abecasis/public/lipids2013/
§SBP	108,039	Sudlow, C. *et al*. 2015 PLOS Medicine	http://biobank.ctsu.ox.ac.uk/crystal/field.cgi?id=4080
§DBP	108,039	Sudlow, C. *et al*. 2015 PLOS Medicine	http://biobank.ctsu.ox.ac.uk/crystal/field.cgi?id=4079
Height	253,288	Wood *et al*. 2014 Naure Genetics	https://www.broadinstitute.org/collaboration/giant/index.php/GIANT_consortium_data_files
EduYears	⌘405,072	Okbay *et al*. 2016 Nature	http://www.thessgac.org/data

Note: *n* = sample size, § effect sizes of genetic instruments on the risk factor were estimated in UK Biobank; ⌘ For EduYears, due to the limited number of SNPs available in the public domain from the meta-analysis of all the samples, we used the summary data from a subset of the data (*n* = 328,917) in the reverse-GSMR analysis.

Supplementary Table 4 Descriptive summary of GWAS data for the 22 common diseases in GERA and UKB

Common disease	Sample size (GERA)		Sample size (UK Biobank)		SNP heritability (GERA)			SNP heritability (UK Biobank)			Genetic correlation			GERA	UK Biobank	
	n_{cases}	$n_{controls}$	n_{cases}	$n_{controls}$	h^2	*s.e.*	*P*	h^2	*s.e.*	*P*	r_g	*s.e.*	*P*	ICD 9	Self-reported	ICD 10
Asthma	10,080	51,767	14,473	97,865	0.13	0.021	1.7E-10	0.16	0.019	1.3E-18	0.75	0.10	3.5E-14	493	asthma	J45-J45
Allergic Rhinitis	15,166	46,681	6,603	105,735	0.05	0.016	1.8E-03	0.09	0.018	3.4E-07	0.84	0.20	4.1E-05	477	hayfever/allergic rhinitis	J30-J30
Cardiovascular Disease	16,399	45,448	14,510	97,828	0.06	0.015	1.1E-05	0.07	0.012	1.0E-09	0.91	0.17	8.7E-08	398, 402, 404, 410, 411, 412, 413, 414, 425, 426, 427, 428, 430, 431, 432, 433, 434, 435, 436, 437, 438, 441	angina, heart attack/myocardial infarction, heart/cardiac problem, irregular heart beat, heart failure/pulmonary odema, atrial fibrillation, atrial flutter, wolff parkinson white / wpw syndrome, sick sinus syndrome, svt / supraventricular tachycardia, hypertrophic cardiomyopathy (hcm / hocm), myocarditis, rheumatic fever, subarachnoid haemorrhage, brain haemorrhage, transient ischaemic attack (tia), subdural haemorrhage/haematoma, cerebral aneurysm, aortic dissection, venous thromboembolic disease, deep venous thrombosis (dvt), cardiomyopathy, aortic aneurysm, aortic aneurysm rupture, ischaemic stroke, heart arrhythmia, cerebrovascular disease	I01, I09, I11, I13, I20, I21, I22, I23, I24, I25, I42, I43, I44, I45, I46, I47, I48, I49, I50, I60, I61, I62, I63, I64, I65, I66, I67, I68, I69, I71
Cancer	18,677	43,170	11,632	100,706	0.09	0.020	6.8E-06	0.03	0.012	5.5E-03	0.92	0.23	6.6E-05	141, 143, 144, 145, 146, 147, 148, 149, 150, 151, 153, 155, 156, 157, 162, 172, 173, 174, 176, 179, 180, 182, 183, 184, 185, 188, 189, 191, 192, 195, 196, 197, 198, 199, 200, 202, 204, 205, 207, 209, 238, 530	/	C01, C03, C04, C05, C06, C07, C08, C09, C10, C11, C12, C13, C14, C15, C16, C18, C22, C23, C24, C25, C33, C43, C44, C45, C46, C47, C48, C49, C50, C53, C54, C55, C56, C57, C58, C59, C60, C61, C64, C67, C71, C72, C76, C77, C78, C79, C80, C81, C82, C83, C84, C85, C91, C92, C94, C96, D48, K22.7

Major Depressive Disorder	7,892	53,955	7,517	104,821	0.08	0.021	1.1E-04	0.08	0.017	3.3E-06	0.94	0.21	6.8E-06	296	depression, post-natal depression	F32, F33
Dermatophytosis	8,428	53,419	27	112,311	0.05	0.021	2.4E-02	/	/	/	/	/	/	110, 111	/	B35, B36
T2D	7,624	54,223	6,024	106,314	0.16	0.024	3.0E-11	0.19	0.022	1.8E-17	0.99	0.11	7.5E-21	250	diabetes, type 2 diabetes	E11
Dyslipidemia	33,024	28,823	16,818	95,520	0.19	0.021	1.1E-18	0.13	0.015	3.0E-17	0.88	0.07	2.7E-38	272	high cholesterol	E78
Hypertensive Disease	31,000	30,847	32,689	79,649	0.22	0.018	6.5E-33	0.20	0.014	1.4E-50	0.98	0.05	2.4E-85	401, 402, 403, 404	essential hypertension, hypertension, gestational hypertension/pre-eclampsia	I10, I11, I12, I13
Hemorrhoids	9,898	51,949	3,826	108,512	0.01	0.019	6.8E-01	0.06	0.024	1.2E-02	/	/	/	455	haemorrhoids / piles	I84
Hernia Abdominopelvic Cavity	6,864	54,983	7,115	105,223	0.03	0.024	1.8E-01	0.06	0.017	9.5E-04	/	/	/	550, 551, 552, 553	hiatus hernia, inguinal hernia, umbilical hernia, femoral hernia, incisional hernia, abdominal hernia	K40, K41, K42, K43
Insomnia	4,346	57,501	979	111,359	0.09	0.029	2.6E-03	0.13	0.068	5.8E-02	/	/	/	307.4, 327, 780.5	insomnia	F51, G47
Iron Deficiency Anemias	2,699	59,148	1,409	110,929	0.07	0.040	8.7E-02	0.04	0.048	4.4E-01	/	/	/	280	iron deficiency anaemia	D50
Irritable Bowel Syndrome	3,359	58,488	3,154	109,184	0.11	0.035	1.1E-03	0.03	0.027	2.2E-01	/	/	/	564	irritable bowel syndrome	K58
Macular Degeneration	4,026	57,821	481	111,857	0.14	0.073	5.2E-02	0.01	0.107	9.2E-01	/	/	/	362	macular degeneration, retinitis pigmentosa	H35
Osteoarthritis	22,022	39,825	11,133	101,205	0.06	0.015	2.8E-05	0.09	0.013	8.9E-14	0.92	0.17	1.4E-07	715	osteoarthritis	M19
Osteoporosis	5,898	55,949	2,084	110,254	0.14	0.028	2.8E-07	0.11	0.033	5.4E-04	0.84	0.23	1.8E-04	733	osteoporosis	M81
Peripheral Vascular Disease	4,708	57,139	1,816	110,522	0.03	0.026	2.2E-01	0.08	0.039	3.3E-02	/	/	/	415, 440, 453	peripheral vascular disease, leg claudication/ intermittent claudication, arterial embolism, pulmonary embolism +/- dvt	I26, I70, I82
Peptic Ulcer	1,004	60,843	2,254	110,084	0.18	0.078	1.9E-02	0.04	0.033	1.8E-01	/	/	/	531, 532, 533, 534	peptic ulcer, duodenal ulcer, gastric/stomach ulcers	K25, K26, K27, K28
Psychiatric Disorder	9,394	52,453	2,564	109,774	0.07	0.020	1.5E-04	0.07	0.030	1.7E-02	0.99	0.31	1.4E-03	292, 295, 296, 297, 298, 300, 307, 309	psychological/psychiatric problem, mania/bipolar disorder/manic depression, schizophrenia, anxiety/panic attacks	F19, F20, F21, F22, F23, F24, F25, F26, F27, F28, F29, F30, F31, F40, F41, F50
Acute Reaction to Stress	4,695	57,152	369	111,969	0.05	0.029	6.2E-02	-0.01	0.128	9.1E-01	/	/	/	308	stress	F43
Varicose Veins	2,711	59,136	2,637	109,701	0.01	0.046	8.3E-01	0.18	0.033	2.9E-08	/	/	/	454	varicose veins	I83

Note: n_{cases} = sample size of cases; n_{controls} = sample size of controls; The SNP-based heritability (h^2_{SNP}) estimated in GERA and UKB are in the liability scale; We only show the genetic correlation (r_g) for diseases that have a nominally significant estimate of h^2_{SNP} in both GERA and UK Biobank (P < 0.05).

Supplementary Table 5 Descriptive summary of GWAS summary data for the 10 common diseases from published case-control studies

Common disease	n_{cases}	$n_{controls}$	Publication	URL	Population prevalence
CAD	60,801	123,504	Nikpey *et al.* 2015 Nautre Genetics	http://www.cardiogramplusc4d.org/data-downloads/	0.064
Rheumatoid Arthritis	14,361	43,923	Okada *et al.* 2014 Nature	http://plaza.umin.ac.jp/~yokada/datasource/software.htm	0.005
Crohn's Disease	5,956	21,770	Liu *et al.* 2015 Nature Genetics	http://www.ibdgenetics.org/downloads.html	0.002
Ulcerative Colitis	6,968	21,770	Liu *et al.* 2015 Nature Genetics	http://www.ibdgenetics.org/downloads.html	0.003
T2D	12,171	56,862	Morris *et al.* 2012 Nature Genetics	http://diagram-consortium.org/downloads.html	0.09
Autism Spectrum Disorder	13,088	16,664	Robinson *et al.* 2016 Nature Genetics	https://www.med.unc.edu/pgc/downloads	0.006
Bipolar Disorder	10,410	10,700	Ruderfer *et al.* 2014 Molecular Psychiatry	https://www.med.unc.edu/pgc/downloads	0.005
Major Depressive Disorder	9,240	9,519	PGC 2013 Molecular Psychiatry	https://www.med.unc.edu/pgc/downloads	0.15
Schizophrenia	36,989	113,075	PGC 2014 Nature	https://www.med.unc.edu/pgc/downloads	0.011
Alzheimer's Disease	17,008	37,154	Lambert *et al.* 2013 Nature Genetics	http://www.pasteur-lille.fr/en/recherche/u744/igap/igap_download.php	0.044
AMD	16,144	17,832	Fritsche *et al.* 2016 Nature Genetics	http://amdgenetics.org/	0.087

Supplementary Table 6 Standard deviations of the risk factors in men and women

Data set	Risk factor (unit)	SD	
		Men	Women
ARIC	BMI (kg/m^2)	3.98	5.49
ARIC	WHRadjBMI	0.04	0.07
ARIC	HDL-c (mmol/L)	0.32	0.44
ARIC	LDL-c (mmol/L)	0.92	1.03
ARIC	TG (mmol/L)	1.13	0.94
UK Biobank	SBP (mmHg)	18.53	20.29
UK Biobank	DBP (mmHg)	10.58	10.57
ARIC	Height (cm)	6.48	5.90
ARIC	EduYears (years)	4.31	3.79

Supplementary Table 7 GSMR estimates of the effects of risk factors on risk factors

**Risk factor (exposure)**	**Risk factor (outcome)**	$\hat{b}_{xy}$	$s.e.(\hat{b}_{xy})$	P_{xy}	n_{SNP}
BMI	WHRadjBMI	-0.02	0.018	3.2E-01	87
BMI	HDL-c	-0.29	0.020	1.1E-48	81
BMI	LDL-c	-0.01	0.020	6.9E-01	85
BMI	TG	0.28	0.019	3.1E-47	86
BMI	SBP	0.06	0.026	1.8E-02	81
BMI	DBP	0.15	0.026	1.0E-08	84
WHRadjBMI	BMI	-0.19	0.018	4.3E-27	44
WHRadjBMI	HDL-c	-0.36	0.025	5.6E-49	39
WHRadjBMI	LDL-c	0.10	0.025	1.1E-04	42
WHRadjBMI	TG	0.36	0.026	2.2E-45	39
WHRadjBMI	SBP	0.10	0.031	1.5E-03	45
WHRadjBMI	DBP	0.12	0.032	1.0E-04	43
HDL-c	BMI	-0.02	0.006	4.1E-03	138
HDL-c	WHRadjBMI	-0.01	0.008	7.0E-02	140
HDL-c	LDL-c	0.00	0.011	9.6E-01	122
HDL-c	TG	-0.22	0.012	< 1.0E-50	95
HDL-c	SBP	-0.03	0.010	7.8E-03	162
HDL-c	DBP	-0.03	0.010	7.8E-03	157
LDL-c	BMI	-0.04	0.006	9.1E-09	135
LDL-c	WHRadjBMI	-0.01	0.007	4.7E-01	141
LDL-c	HDL-c	-0.11	0.009	8.7E-38	116
LDL-c	TG	0.20	0.011	< 1.0E-50	100
LDL-c	SBP	0.02	0.010	1.0E-01	143
LDL-c	DBP	-0.02	0.010	1.4E-02	139
TG	BMI	-0.02	0.007	5.1E-03	86
TG	WHRadjBMI	0.05	0.009	5.7E-07	93
TG	HDL-c	-0.48	0.014	< 1.0E-50	57
TG	LDL-c	0.29	0.013	< 1.0E-50	71
TG	SBP	0.02	0.012	1.6E-01	100
TG	DBP	0.01	0.013	2.7E-01	99
SBP	BMI	-0.08	0.021	8.8E-05	24
SBP	WHRadjBMI	0.01	0.025	7.4E-01	26
SBP	HDL-c	0.04	0.028	1.6E-01	26
SBP	LDL-c	-0.06	0.029	5.2E-02	26
SBP	TG	0.01	0.029	7.8E-01	26
SBP	DBP	0.61	0.034	< 1.0E-50	28
DBP	BMI	-0.08	0.020	4.2E-05	26
DBP	WHRadjBMI	0.02	0.025	4.4E-01	27
DBP	HDL-c	-0.03	0.031	3.1E-01	27
DBP	LDL-c	-0.04	0.033	2.0E-01	27
DBP	TG	0.08	0.030	1.1E-02	28
DBP	SBP	0.66	0.031	< 1.0E-50	30

Supplementary Table 8 Estimates of SNP-based heritability for the risk factors and genetic correlation between the risk factors

**Risk factor**	BMI	WHRadjBMI	HDL-c	LDL-c	TG	SBP	DBP
BMI	0.14	-0.08	-0.27	0.02	0.19	0.10	0.20
WHRadjBMI	-0.08	0.09	-0.24	0.12	0.31	0.14	0.11
HDL-c	-0.27	-0.24	0.24	-0.07	-0.60	-0.09	-0.14
LDL-c	0.02	0.12	-0.07	0.20	0.36	0.00	0.02
TG	0.19	0.31	-0.60	0.36	0.27	0.13	0.12
SBP	0.10	0.14	-0.09	0.00	0.13	0.12	0.65
DBP	0.20	0.11	-0.14	0.02	0.12	0.65	0.14

Note: The diagonal elements are the estimates of SNP-based heritability, and the off-diagonal elements are the estimates of genetic correlation.

Supplementary Table 9 Estimated regression intercepts from the bivariate LDSC analysis between pairwise risk factors

Risk factor	Risk factor	$\hat{b}_0$	*s.e.*	*p*-value
BMI	HDL-c	-0.109	0.006	2.34E-79
	LDL-c	0.043	0.006	5.87E-13
	TG	0.106	0.006	6.63E-80
	SBP	0.004	0.006	4.61E-01
HDL-c	BMI	-0.109	0.006	2.34E-79
	LDL-c	-0.088	0.014	3.79E-10
	TG	-0.279	0.020	3.15E-44
	SBP	0.005	0.005	3.64E-01
LDL-c	BMI	0.043	0.006	5.87E-13
	HDL-c	-0.088	0.014	3.79E-10
	TG	0.166	0.011	2.45E-51
	SBP	0.000	0.005	1.00E+00
TG	BMI	0.106	0.006	6.63E-80
	HDL-c	-0.279	0.020	3.15E-44
	LDL-c	0.166	0.011	2.45E-51
	SBP	-0.006	0.006	2.76E-01
SBP	BMI	0.004	0.006	4.61E-01
	HDL-c	0.005	0.005	3.64E-01
	LDL-c	0.000	0.005	1.00E+00
	TG	-0.006	0.006	2.76E-01

Note: $\hat{b}_0$ = the regression intercept from the bivariate LDSC analysis.

Supplementary Table 10 Estimated regression intercepts from the bivariate LDSC analysis between risk factors and diseases

Data set	Phenotype	BMI	HDL-c	LDL-c	TG	SBP
Community-based study	Asthma	-0.001	0.000	0.001	-0.006	0.002
	Cardiovascular Disease	-0.005	0.003	0.010	0.000	-0.030
	Dermatophytosis	0.004	-0.009	0.004	0.007	0.007
	T2D	-0.004	0.005	0.005	-0.003	0.005
	Dyslipidemia	-0.009	-0.002	0.011	-0.024	0.019
	Hypertensive Disease	-0.004	0.003	0.008	-0.001	0.209
	Osteoarthritis	-0.005	0.004	0.002	0.004	-0.003
	Osteoporosis	0.002	0.004	-0.011	-0.009	-0.002
	Peripheral Vascular Disease	-0.001	-0.002	-0.006	-0.006	-0.001
	Disease Count	-0.007	0.007	0.002	-0.012	0.054
Case-control study	CAD	0.008	-0.012	0.022	0.009	0.005
	T2D	0.056	-0.036	-0.004	0.046	0.002
	AMD	0.003	-0.003	-0.007	0.003	0.003

Supplementary Table 11 Estimated causative effects of 5 risk factors on common diseases from the conditional GSMR analysis

Risk factor	Common disease	Covariates	Method	OR	95%CI Lower Bound	95%CI Upper Bound	$\hat{b}_{xy}$	$s.e.(\hat{b}_{xy})$	P_{xy}	n_{SNP}
BMI	Asthma (Community)	HDL-c, LDL-c, TG, SBP	Original	1.35	1.20	1.51	0.300	0.059	3.75E-07	83
			Conditional	1.31	1.17	1.48	0.273	0.060	5.20E-06	86
BMI	Cardiovascular Disease	HDL-c, LDL-c, TG, SBP	Original	1.30	1.16	1.45	0.260	0.056	4.16E-06	84
			Conditional	1.22	1.09	1.37	0.199	0.059	7.00E-04	87
BMI	CAD (Case-control)	HDL-c, LDL-c, TG, SBP	Original	1.70	1.53	1.89	0.532	0.054	2.74E-23	86
			Conditional	1.51	1.35	1.69	0.413	0.056	2.62E-13	89
BMI	Dermatophytosis (Community)	HDL-c, LDL-c, TG, SBP	Original	1.67	1.36	2.03	0.511	0.102	5.55E-07	86
			Conditional	1.65	1.35	2.03	0.503	0.104	1.46E-06	87
BMI	T2D (Community)	HDL-c, LDL-c, TG, SBP	Original	3.29	2.82	3.83	1.190	0.078	<1.0E-50	80
			Conditional	3.24	2.78	3.79	1.176	0.079	6.23E-50	82
BMI	T2D (Case-control)	HDL-c, LDL-c, TG, SBP	Original	3.12	2.56	3.81	1.139	0.102	4.37E-29	89
			Conditional	2.75	2.24	3.38	1.011	0.105	8.05E-22	90
BMI	Dyslipidemia (Community)	HDL-c, LDL-c, TG, SBP	Original	1.37	1.23	1.53	0.317	0.054	4.78E-09	76
			Conditional	1.19	1.06	1.34	0.174	0.059	3.17E-03	85
BMI	Hypertensive Disease	HDL-c, LDL-c, TG, SBP	Original	1.85	1.69	2.02	0.614	0.047	9.48E-40	78
			Conditional	1.53	1.38	1.71	0.428	0.054	2.86E-15	85
BMI	Osteoarthritis (Community)	HDL-c, LDL-c, TG, SBP	Original	1.50	1.34	1.68	0.405	0.058	4.08E-12	81
			Conditional	1.53	1.36	1.71	0.422	0.060	1.30E-12	84
BMI	Osteoporosis (Community)	HDL-c, LDL-c, TG, SBP	Original	0.68	0.55	0.83	-0.387	0.104	2.06E-04	84
			Conditional	0.68	0.56	0.84	-0.378	0.106	3.60E-04	87
BMI	Peripheral Vascular Disease	HDL-c, LDL-c, TG, SBP	Original	1.59	1.28	1.98	0.465	0.111	2.93E-05	84
			Conditional	1.60	1.28	1.99	0.468	0.113	3.51E-05	87
BMI	Disease Count (Community)	HDL-c, LDL-c, TG, SBP	Original	1.51	1.42	1.60	0.412	0.030	1.31E-41	81
			Conditional	1.43	1.34	1.52	0.359	0.032	4.22E-29	84
HDL-c	Cardiovascular Disease	BMI, LDL-c, TG, SBP	Original	0.88	0.85	0.93	-0.122	0.023	9.60E-08	151
			Conditional	0.96	0.92	1.01	-0.036	0.025	1.53E-01	127
HDL-c	CAD (Case-control)	BMI, LDL-c, TG, SBP	Original	0.84	0.80	0.88	-0.178	0.023	2.38E-14	150
			Conditional	1.00	0.95	1.05	-0.005	0.026	8.57E-01	124
HDL-c	T2D (Community)	BMI, LDL-c, TG, SBP	Original	0.83	0.78	0.88	-0.185	0.031	3.33E-09	143
			Conditional	0.88	0.82	0.95	-0.126	0.036	4.47E-04	114
HDL-c	T2D (Case-control)	BMI, LDL-c, TG, SBP	Original	0.81	0.74	0.89	-0.206	0.048	1.56E-05	152
			Conditional	0.94	0.85	1.04	-0.063	0.052	2.27E-01	123
HDL-c	Dyslipidemia (Community)	BMI, LDL-c, TG, SBP	Original	0.81	0.77	0.85	-0.213	0.024	2.63E-18	121
			Conditional	1.01	0.96	1.06	0.013	0.025	6.13E-01	125
HDL-c	Hypertensive Disease	BMI, LDL-c, TG, SBP	Original	0.88	0.85	0.91	-0.132	0.018	1.69E-13	147
			Conditional	0.94	0.90	0.99	-0.058	0.023	1.09E-02	126
HDL-c	Disease Count (Community)	BMI, LDL-c, TG, SBP	Original	0.94	0.91	0.96	-0.065	0.013	3.01E-07	141
			Conditional	0.97	0.94	1.00	-0.030	0.014	3.53E-02	119
HDL-c	AMD (Case-control)	BMI, LDL-c, TG, SBP	Original	1.36	1.26	1.46	0.307	0.038	5.89E-16	150
			Conditional	1.36	1.25	1.48	0.309	0.043	5.12E-13	117
LDL-c	Cardiovascular Disease	BMI, HDL-c, TG, SBP	Original	1.22	1.16	1.27	0.195	0.022	1.06E-18	138
			Conditional	1.16	1.11	1.21	0.149	0.023	9.65E-11	110
LDL-c	CAD (Case-control)	BMI, HDL-c, TG, SBP	Original	1.50	1.44	1.57	0.408	0.022	<1.0E-50	138
			Conditional	1.60	1.52	1.68	0.470	0.025	< 1.0E-50	108
LDL-c	Dyslipidemia (Community)	BMI, HDL-c, TG, SBP	Original	3.36	3.22	3.51	1.212	0.022	<1.0E-50	127
			Conditional	3.35	3.19	3.51	1.208	0.025	< 1.0E-50	110
LDL-c	Disease Count (Community)	BMI, HDL-c, TG, SBP	Original	1.21	1.18	1.24	0.189	0.012	<1.0E-50	132
			Conditional	1.22	1.19	1.25	0.201	0.012	< 1.0E-50	111
LDL-c	T2D (Case-control)	BMI, HDL-c, TG, SBP	Original	0.84	0.77	0.92	-0.171	0.044	1.05E-04	138
			Conditional	0.84	0.77	0.92	-0.173	0.045	1.14E-04	111
TG	Cardiovascular Disease	BMI, HDL-c, LDL-c, SBP	Original	1.14	1.08	1.21	0.135	0.029	2.46E-06	95
			Conditional	1.08	1.01	1.14	0.073	0.031	1.81E-02	96
TG	CAD (Case-control)	BMI, HDL-c, LDL-c, SBP	Original	1.33	1.26	1.40	0.284	0.028	4.71E-25	96
			Conditional	1.15	1.09	1.22	0.140	0.030	2.42E-06	96

TG	Dyslipidemia (Community)	BMI, HDL-c, LDL-c, SBP	Original	2.09	1.98	2.21	0.736	0.028	<1.0E-50	85
			Conditional	1.60	1.50	1.70	0.469	0.032	3.40E-48	96
TG	Hypertensive Disease	BMI, HDL-c, LDL-c, SBP	Original	1.24	1.18	1.29	0.211	0.023	1.56E-20	94
			Conditional	1.17	1.11	1.24	0.157	0.028	1.34E-08	99
TG	Disease Count (Community)	BMI, HDL-c, LDL-c, SBP	Original	1.19	1.15	1.22	0.170	0.016	3.05E-27	91
			Conditional	1.14	1.10	1.18	0.128	0.018	3.49E-13	94
SBP	Cardiovascular Disease	BMI, HDL-c, LDL-c, TG	Original	1.40	1.22	1.61	0.339	0.070	1.30E-06	28
			Conditional	1.48	1.28	1.71	0.392	0.075	1.72E-07	26
SBP	CAD (Case-control)	BMI, HDL-c, LDL-c, TG	Original	1.73	1.51	1.98	0.547	0.069	1.42E-15	27
			Conditional	1.62	1.39	1.88	0.483	0.077	3.19E-10	22
SBP	Dyslipidemia (Community)	BMI, HDL-c, LDL-c, TG	Original	1.50	1.33	1.71	0.408	0.065	2.61E-10	28
			Conditional	1.72	1.48	2.00	0.541	0.077	1.65E-12	26
SBP	Hypertensive Disease	BMI, HDL-c, LDL-c, TG	Original	4.38	3.84	5.00	1.477	0.067	<1.0E-50	25
			Conditional	4.67	4.05	5.38	1.541	0.072	< 1.0E-50	24
SBP	Disease Count (Community)	BMI, HDL-c, LDL-c, TG	Original	1.43	1.33	1.54	0.357	0.038	3.32E-21	28
			Conditional	1.51	1.40	1.64	0.415	0.041	1.50E-23	26

Note: Highlighted are the significant associations (P_{GSMR} < 2.2e−4) in the conditional GSMR analysis; Original - GSMR analysis (see all the results from GSMR in **Supplementary Data #1**); Conditional - conditional GSMR analysis; OR = $\exp(\hat{b}_{xy})$; Lower bound and upper bound represent the 95% confidence interval of OR; b_{xy} = the estimated effect of risk factor on disease by GSMR; $s.e.(\hat{b}_{xy})$= standard error of the estimate of $\hat{b}_{xy}$; P_{xy} = p-value for $\hat{b}_{xy}$; n_{SNP} = the number of genetic instruments used in the analysis.

Supplementary Table 12 Causative effects of height and educational attainment on common diseases estimated by GSMR

Source	Risk factor	Common disease	OR	95%CI Lower Bound	95%CI Upper Bound	$\hat{b}_{xy}$	$s.e.(\hat{b}_{xy})$	P_{xy}	n_{SNP}
Community-based study	Height	Asthma	0.90	0.87	0.93	-0.109	0.017	3.49E-10	790
	Height	Allergic Rhinitis	0.96	0.92	0.99	-0.044	0.019	2.12E-02	807
	Height	Cardiovascular Disease	1.07	1.04	1.11	0.071	0.017	2.73E-05	799
	Height	Cancer	1.09	1.06	1.13	0.087	0.017	3.15E-07	799
	Height	Major Depressive Disorder	1.00	0.96	1.04	0.000	0.021	9.90E-01	807
	Height	Dermatophytosis	1.24	1.17	1.32	0.215	0.030	1.50E-12	813
	Height	T2D	0.86	0.83	0.90	-0.146	0.023	4.25E-10	792
	Height	Dyslipidemia	0.84	0.81	0.86	-0.176	0.015	2.07E-30	780
	Height	Hypertensive Disease	0.86	0.84	0.88	-0.150	0.014	5.44E-28	776
	Height	Hemorrhoids	1.04	0.99	1.08	0.036	0.023	1.24E-01	807
	Height	Hernia Abdominopelvic Cavity	0.93	0.89	0.97	-0.076	0.023	1.04E-03	794
	Height	Insomnia	1.01	0.94	1.09	0.013	0.036	7.09E-01	808
	Height	Iron Deficiency Anemias	1.05	0.97	1.13	0.046	0.040	2.54E-01	808
	Height	Irritable Bowel Syndrome	0.82	0.77	0.87	-0.204	0.032	1.24E-10	804
	Height	Macular Degeneration	0.96	0.89	1.05	-0.037	0.042	3.74E-01	805
	Height	Osteoarthritis	1.09	1.05	1.12	0.083	0.017	1.83E-06	797
	Height	Osteoporosis	0.85	0.80	0.91	-0.161	0.031	3.27E-07	794
	Height	Peripheral Vascular Disease	1.15	1.08	1.23	0.141	0.033	1.96E-05	808
	Height	Peptic Ulcer	0.97	0.89	1.06	-0.032	0.044	4.69E-01	797
	Height	Psychiatric Disorder	0.99	0.94	1.04	-0.010	0.025	6.86E-01	803
	Height	Acute Reaction to Stress	0.85	0.79	0.92	-0.158	0.038	2.66E-05	807
	Height	Varicose Veins	1.38	1.29	1.48	0.325	0.035	1.33E-20	803
	Height	Disease Count	0.99	0.97	1.01	-0.010	0.009	2.64E-01	788
	EduYears	Asthma	0.93	0.81	1.06	-0.073	0.068	2.83E-01	118
	EduYears	Allergic Rhinitis	1.09	0.94	1.25	0.082	0.074	2.70E-01	119
	EduYears	Cardiovascular Disease	0.73	0.64	0.83	-0.311	0.066	2.48E-06	119
	EduYears	Cancer	0.89	0.78	1.01	-0.121	0.068	7.40E-02	117
	EduYears	Major Depressive Disorder	0.83	0.70	0.98	-0.188	0.083	2.42E-02	119
	EduYears	Dermatophytosis	1.34	1.07	1.68	0.291	0.116	1.23E-02	126
	EduYears	T2D	0.64	0.53	0.76	-0.451	0.089	4.47E-07	119
	EduYears	Dyslipidemia	0.71	0.63	0.80	-0.338	0.060	1.66E-08	119
	EduYears	Hypertensive Disease	0.62	0.56	0.69	-0.476	0.052	4.79E-20	118
	EduYears	Hemorrhoids	0.89	0.75	1.07	-0.112	0.091	2.18E-01	119
	EduYears	Hernia Abdominopelvic Cavity	0.80	0.68	0.96	-0.218	0.089	1.42E-02	118
	EduYears	Insomnia	0.88	0.60	1.30	-0.125	0.196	5.25E-01	119
	EduYears	Iron Deficiency Anemias	0.74	0.50	1.09	-0.307	0.200	1.24E-01	117
	EduYears	Irritable Bowel Syndrome	0.72	0.57	0.93	-0.322	0.125	1.01E-02	119
	EduYears	Macular Degeneration	1.26	0.79	1.99	0.230	0.235	3.28E-01	119
	EduYears	Osteoarthritis	0.84	0.73	0.95	-0.180	0.067	7.14E-03	119
	EduYears	Osteoporosis	1.17	0.92	1.48	0.154	0.122	2.08E-01	119
	EduYears	Peripheral Vascular Disease	0.54	0.38	0.75	-0.624	0.169	2.12E-04	119
	EduYears	Peptic Ulcer	0.62	0.44	0.87	-0.477	0.174	5.99E-03	119
	EduYears	Psychiatric Disorder	0.86	0.71	1.05	-0.147	0.098	1.33E-01	118
	EduYears	Acute Reaction to Stress	0.58	0.40	0.82	-0.551	0.181	2.31E-03	119
	EduYears	Varicose Veins	0.88	0.67	1.15	-0.131	0.138	3.42E-01	118
	EduYears	Disease Count	0.74	0.70	0.80	-0.296	0.035	1.45E-17	119
Case-control study	Height	CAD	0.77	0.74	0.79	-0.265	0.015	1.53E-66	825
	Height	Rheumatoid Arthritis	1.29	1.22	1.37	0.257	0.030	3.55E-17	813
	Height	Crohn's Disease	1.06	0.99	1.15	0.060	0.038	1.16E-01	827
	Height	Ulcerative Colitis	0.89	0.83	0.95	-0.117	0.036	1.12E-03	825
	Height	T2D	0.84	0.79	0.89	-0.171	0.031	2.59E-08	825
	Height	Autism Spectrum Disorder	1.04	0.95	1.13	0.035	0.046	4.49E-01	845
	Height	Bipolar Disorder	1.00	0.93	1.07	-0.001	0.037	9.75E-01	760
	Height	Major Depressive Disorder	0.91	0.85	0.98	-0.095	0.037	1.01E-02	768

	Height	Schizophrenia	1.00	0.96	1.03	-0.002	0.018	9.07E-01	808
	Height	Alzheimer's Disease	0.85	0.81	0.89	-0.164	0.027	6.00E-10	822
	Height	AMD	0.91	0.86	0.96	-0.096	0.026	1.77E-04	827
	EduYears	CAD	0.63	0.56	0.71	-0.467	0.060	8.12E-15	128
	EduYears	Rheumatoid Arthritis	0.44	0.35	0.56	-0.818	0.121	1.49E-11	115
	EduYears	Crohn's Disease	1.09	0.81	1.46	0.083	0.150	5.80E-01	124
	EduYears	Ulcerative Colitis	0.89	0.68	1.16	-0.119	0.139	3.89E-01	125
	EduYears	T2D	0.79	0.55	1.15	-0.232	0.188	2.18E-01	53
	EduYears	Autism Spectrum Disorder	2.30	1.62	3.27	0.834	0.179	2.98E-06	126
	EduYears	Bipolar Disorder	1.29	0.77	2.17	0.258	0.263	3.28E-01	35
	EduYears	Major Depressive Disorder	0.69	0.41	1.16	-0.376	0.266	1.58E-01	35
	EduYears	Schizophrenia	0.99	0.86	1.15	-0.007	0.073	9.26E-01	113
	EduYears	Alzheimer's Disease	0.61	0.50	0.76	-0.487	0.108	6.55E-06	114
	EduYears	AMD	0.99	0.82	1.20	-0.008	0.097	9.37E-01	129

Note: Highlighted are the significant associations (P_{GSMR} < 7.6e–4). OR = $\exp(\hat{b}_{xy})$; Lower bound and upper bound represent the 95% confidence interval of OR; $\hat{b}_{xy}$ = the estimated effect of risk factor on disease by GSMR; $s.e.(\hat{b}_{xy})$ = standard error of the estimate of $\hat{b}_{xy}$; P_{xy} = p-value for $\hat{b}_{xy}$; n_{SNP} = the number of genetic instruments used in the GSMR analysis.

Supplementary Table 13 Estimated causative effects of common diseases on risk factors from the reverse-GSMR analysis

Source	Risk factor	Common Disease	GSMR analysis				Reverse-GSMR analysis			
			$\hat{b}_{xy}$	$s.e.(\hat{b}_{xy})$	P_{xy}	n_{SNP}	$\hat{b}_{xy}$	$s.e.(\hat{b}_{xy})$	P_{xy}	n_{SNP}
Community -based study	BMI	Asthma	0.300	0.059	3.75E-07	83	-0.009	0.008	2.48E-01	28
	BMI	T2D	1.190	0.078	2.17E-52	80	-0.065	0.006	3.60E-26	17
	BMI	Dyslipidemia	0.317	0.054	4.78E-09	76	-0.033	0.005	2.01E-10	51
	BMI	Hypertensive Disease	0.614	0.047	9.48E-40	78	-0.024	0.008	3.42E-03	46
	BMI	Osteoporosis	-0.387	0.104	2.06E-04	84	-0.003	0.008	7.41E-01	10
	WHRadjBMI	T2D	0.469	0.105	7.49E-06	38	0.044	0.007	5.01E-10	20
	WHRadjBMI	Dyslipidemia	0.380	0.067	1.16E-08	42	0.020	0.006	1.84E-03	53
	WHRadjBMI	Hypertensive Disease	0.378	0.062	7.93E-10	39	0.012	0.009	2.13E-01	52
	HDL-c	T2D	-0.185	0.031	3.33E-09	143	-0.003	0.008	7.43E-01	16
	HDL-c	Dyslipidemia	-0.213	0.024	2.63E-18	121	-0.108	0.009	7.69E-35	40
	HDL-c	Hypertensive Disease	-0.132	0.018	1.69E-13	147	-0.016	0.011	1.51E-01	51
	LDL-c	Dyslipidemia	1.212	0.022	0.00E+00	127	0.545	0.011	0.00E+00	43
	TG	Dyslipidemia	0.736	0.028	1.74E-151	85	0.144	0.011	1.62E-37	30
	TG	Hypertensive Disease	0.211	0.023	1.56E-20	94	0.000	0.011	9.66E-01	50
	SBP	Dyslipidemia	0.408	0.065	2.61E-10	28	0.013	0.008	8.52E-02	65
	SBP	Hypertensive Disease	1.477	0.067	1.55E-107	25	0.340	0.011	5.49E-210	58
	SBP	Disease Count	0.357	0.038	3.32E-21	28	0.093	0.041	2.42E-02	13
	DBP	Dyslipidemia	0.268	0.059	5.40E-06	29	-0.020	0.008	1.10E-02	65
	DBP	Hypertensive Disease	1.171	0.060	8.60E-85	27	0.315	0.011	1.40E-178	58
	DBP	Disease Count	0.225	0.033	1.55E-11	29	0.099	0.041	1.68E-02	13
	Height	Asthma	-0.109	0.017	3.49E-10	790	-0.021	0.008	1.01E-02	24
	Height	Cancer	0.087	0.017	3.15E-07	799	-0.037	0.014	9.39E-03	12
	Height	T2D	-0.146	0.023	4.25E-10	792	0.006	0.006	3.80E-01	18
	Height	Dyslipidemia	-0.176	0.015	2.07E-30	780	-0.033	0.006	3.32E-08	43
	Height	Hypertensive Disease	-0.150	0.014	5.44E-28	776	0.008	0.009	3.45E-01	40
	EduYears	T2D	-0.451	0.089	4.47E-07	119	0.000	0.005	9.64E-01	23
	EduYears	Dyslipidemia	-0.338	0.060	1.66E-08	119	0.002	0.004	6.42E-01	55
	EduYears	Hypertensive Disease	-0.476	0.052	4.79E-20	118	-0.003	0.007	6.50E-01	48
	EduYears	Disease Count	-0.296	0.035	1.45E-17	119	-0.111	0.025	1.01E-05	11
Case-control study	BMI	CAD	0.532	0.054	2.74E-23	86	-0.013	0.006	3.68E-02	38
	BMI	T2D	1.139	0.102	4.37E-29	89	-0.044	0.006	3.74E-15	11
	WHRadjBMI	CAD	0.316	0.065	1.33E-06	44	0.003	0.007	7.08E-01	40
	WHRadjBMI	T2D	0.597	0.127	2.84E-06	45	0.045	0.007	5.49E-12	12
	HDL-c	CAD	-0.178	0.023	2.38E-14	150	-0.004	0.009	6.55E-01	38
	HDL-c	T2D	-0.206	0.048	1.56E-05	152	-0.011	0.007	1.26E-01	11
	HDL-c	AMD	0.307	0.038	5.89E-16	150	0.004	0.003	1.76E-01	64
	LDL-c	CAD	0.408	0.022	1.48E-73	138	-0.027	0.009	3.61E-03	37
	LDL-c	T2D	-0.171	0.044	1.05E-04	138	0.010	0.007	1.44E-01	13
	TG	CAD	0.284	0.028	4.71E-25	96	0.002	0.009	7.83E-01	41
	SBP	CAD	0.547	0.069	1.42E-15	27	0.033	0.010	4.86E-04	45
	DBP	CAD	0.310	0.072	1.58E-05	26	0.026	0.010	8.74E-03	41
	Height	CAD	-0.265	0.015	1.53E-66	825	0.007	0.007	2.99E-01	33
	Height	Rheumatoid Arthritis	0.257	0.030	3.55E-17	813	-0.001	0.002	7.80E-01	71
	Height	T2D	-0.171	0.031	2.59E-08	825	0.004	0.006	5.15E-01	11
	Height	Alzheimer's Disease	-0.164	0.027	6.00E-10	822	0.005	0.003	1.43E-01	24
	Height	AMD	-0.096	0.026	1.77E-04	827	0.005	0.002	5.99E-03	60
	EduYears	CAD	-0.467	0.060	8.12E-15	128	0.006	0.006	2.82E-01	41
	EduYears	Rheumatoid Arthritis	-0.818	0.121	1.49E-11	115	-0.001	0.002	6.47E-01	91
	EduYears	Alzheimer's Disease	-0.487	0.108	6.55E-06	114	0.008	0.002	9.21E-04	29

Note: The reverse-GSMR analysis was limited the diseases with at least 10 instruments (**Supplementary Note 7**); Highlighted are the significant associations ($P_{\text{reverse-GSMR}}$ < 1.0e–3).

Supplementary References

1. Bowden J, Davey Smith G, Burgess S. Mendelian randomization with invalid instruments: effect estimation and bias detection through Egger regression. *Int J Epidemiol* **44**, 512-525 (2015).

2. Burgess S, Butterworth A, Thompson SG. Mendelian Randomization Analysis With Multiple Genetic Variants Using Summarized Data. *Genet Epidemiol* **37**, 658-665 (2013).

3. Burgess S, Dudbridge F, Thompson SG. Combining information on multiple instrumental variables in Mendelian randomization: comparison of allele score and summarized data methods. *Stat Med* **35**, 1880-1906 (2016).

4. Pickrell JK, Berisa T, Liu JZ, Segurel L, Tung JY, Hinds DA. Detection and interpretation of shared genetic influences on 42 human traits. *Nat Genet* **48**, 709-717 (2016).

5. Aschard H, Vilhjalmsson BJ, Joshi AD, Price AL, Kraft P. Adjusting for Heritable Covariates Can Bias Effect Estimates in Genome-Wide Association Studies. *Am J Hum Genet* **96**, 329-339 (2015).

6. Fahrmeir L, Kaufmann H. Consistency and Asymptotic Normality of the Maximum-Likelihood Estimator in Generalized Linear-Models. *Ann Stat* **13**, 342-368 (1985).

7. Locke AE*, et al.* Genetic studies of body mass index yield new insights for obesity biology. *Nature* **518**, 197-206 (2015).

8. Shungin D*, et al.* New genetic loci link adipose and insulin biology to body fat distribution. *Nature* **518**, 187-196 (2015).

9. Njolstad I, Arnesen E, Lund-Larsen PG. Body height, cardiovascular risk factors, and risk of stroke in middle-aged men and women. A 14-year follow-up of the Finnmark Study. *Circulation* **94**, 2877-2882 (1996).

10. Wannamethee SG, Shaper AG, Whincup PH, Walker M. Adult height, stroke, and coronary heart disease. *Am J Epidemiol* **148**, 1069-1076 (1998).

11. Emerging Risk Factors Collaboration. Adult height and the risk of cause-specific death and vascular morbidity in 1 million people: individual participant meta-analysis. *Int J Epidemiol* **41**, 1419-1433 (2012).

12. Nuesch E*, et al.* Adult height, coronary heart disease and stroke: a multi-locus Mendelian randomization meta-analysis. *Int J Epidemiol* **45**, 1927-1937 (2015).

13. Green J*, et al.* Height and cancer incidence in the Million Women Study: prospective cohort, and meta-analysis of prospective studies of height and total cancer risk. *Lancet Oncol* **12**, 785-794 (2011).

14. Thrift AP*, et al.* Mendelian randomization study of height and risk of colorectal cancer. *Int J Epidemiol* **44**, 662-672 (2015).

15. Rutter M. Cognitive Deficits in the Pathogenesis of Autism. *J Child Psychol Psychiatry* **24**, 513-531 (1983).

16. McGrath JJ, Wray NR, Pedersen CB, Mortensen PB, Greve AN, Petersen L. The association between family history of mental disorders and general cognitive ability. *Transl Psychiatry* **4**, e412 (2014).

17. Calle EE, Thun MJ, Petrelli JM, Rodriguez C, Heath CW, Jr. Body-mass index and mortality in a prospective cohort of U.S. adults. *N Engl J Med* **341**, 1097-1105 (1999).

18. Bulik-Sullivan B*, et al.* An atlas of genetic correlations across human diseases and traits. *Nat Genet* **47**, 1236-1241 (2015).

19. Bulik-Sullivan BK*, et al.* LD Score regression distinguishes confounding from polygenicity in genome-wide association studies. *Nat Genet* **47**, 291-295 (2015).

20. Clark PM, Kunkel M, Monos DS. The dichotomy between disease phenotype databases and the implications for understanding complex diseases involving the major histocompatibility complex. *Int J Immunogenet* **42**, 413-422 (2015).
