# Supplementary material for: Causal associations between risk factors and common diseases inferred from GWAS summary data
Source: Nat Commun. 2018 Jan 15;9:224. doi: 10.1038/s41467-017-02317-2 (PMC5768719; doi:10.1038/s41467-017-02317-2)
Supplement: Supplementary file 3 — Description of Additional Supplementary Files [file 41467_2017_2317_MOESM3_ESM.pdf]

Description of Additional Supplementary Files

File Name: Supplementary Data 1
Description: The effects of 7 risk factors on common diseases estimated by GSMR.

File Name: Supplementary Data 2
Description: The effects of 7 risk factors on common diseases estimated by other methods.
